# SETDB1 modulates the TGFβ response in Duchenne muscular dystrophy myotubes

**DOI:** 10.1126/sciadv.adj8042

**Published:** 2024-05-01

**Authors:** Alice Granados, Maeva Zamperoni, Roberta Rapone, Maryline Moulin, Ekaterina Boyarchuk, Costas Bouyioukos, Laurence Del Maestro, Véronique Joliot, Elisa Negroni, Myriame Mohamed, Sandra Piquet, Anne Bigot, Fabien Le Grand, Sonia Albini, Slimane Ait-Si-Ali

**Affiliations:** ^1^ Université Paris Cité, CNRS, Epigenetics and Cell Fate, UMR7216, F-75013 Paris, France.; ^2^ Sorbonne Université, Inserm, Institut de Myologie, Centre de Recherche en Myologie, Paris, France.; ^3^ Université Claude Bernard Lyon 1, CNRS UMR 5261, INSERM U1315, Institut NeuroMyoGène, Pathophysiology and Genetics of Neuron and Muscle (PGNM) Unit, 69008 Lyon, France.

## Abstract

Overactivation of the transforming growth factor-β (TGFβ) signaling in Duchenne muscular dystrophy (DMD) is a major hallmark of disease progression, leading to fibrosis and muscle dysfunction. Here, we investigated the role of SETDB1 (SET domain, bifurcated 1), a histone lysine methyltransferase involved in muscle differentiation. Our data show that, following TGFβ induction, SETDB1 accumulates in the nuclei of healthy myotubes while being already present in the nuclei of DMD myotubes where TGFβ signaling is constitutively activated. Transcriptomics revealed that depletion of SETDB1 in DMD myotubes leads to down-regulation of TGFβ target genes coding for secreted factors involved in extracellular matrix remodeling and inflammation. Consequently, SETDB1 silencing in DMD myotubes abrogates the deleterious effect of their secretome on myoblast differentiation by impairing myoblast pro-fibrotic response. Our findings indicate that SETDB1 potentiates the TGFβ–driven fibrotic response in DMD muscles, providing an additional axis for therapeutic intervention.

## INTRODUCTION

Histone lysine methylation/demethylation is a major epigenetic mechanism that controls chromatin states (*1*), with lysine 9 of histone 3 trimethylation (H3K9me3) being a hallmark of repressed chromatin (*2*). SETDB1 (SET domain, bifurcated 1, also called KMT1E, or ESET in the mouse) (*3*) is one of the H3K9 SUV39 KMT (lysine methyltransferase) family members. SETDB1 regulates many cellular states, such as stemness and terminal differentiation, including skeletal muscle terminal differentiation (*1*, *3*). Although SETDB1 is mainly nuclear in proliferating myoblasts, where it represses terminal differentiation genes through H3K9me3 deposition, we have uncovered an original mechanism whereby murine Setdb1 is exported to the cytoplasm of differentiating myoblasts in a Wnt-mediated manner, allowing the derepression of late differentiation genes and the formation of mature multinucleated myotubes (*4*). SETDB1 is associated with many human diseases including inflammatory bowel disease (*5*), many cancer types (*6*), and neuropsychiatric, genetic, and cardiovascular diseases (*3*).

Dystrophinopathies are a spectrum of muscle genetic diseases caused by alterations in the *Dystrophin* gene, and Duchenne muscular dystrophy (DMD) is the most severe form caused by the total absence of the coded protein (*7*, *8*). Dystrophin is essential for the maintenance of muscle membrane integrity by providing a strong mechanical link between the actin cytoskeleton and the extracellular matrix (ECM) (*9*, *10*). Thus, the lack of functional dystrophin is deleterious to membrane integrity, causing continuous degeneration of skeletal muscles (*11*), leading to exhaustion of muscle stem cells (MuSCs), progressive muscle wasting, chronic inflammation, and severe fibrosis (i.e., accumulation of ECM components such as collagens), which is the most prominent feature of DMD. Fibrosis is defined as pathological wound healing with an abnormal deposition of ECM resulting in the replacement of functional tissue by fibrotic connective tissue, which leads to an alteration of tissue and organ homeostasis (*12*, *13*). Fibrosis involves intracellular and extracellular soluble effectors also called secretome, including pro- and anti-inflammatory cytokines, growth factors, and ECM remodelers such as TGFβ, matrix metalloproteinases (MMP)/tissue inhibitors of MMPs (TIMPs), and collagens (*14*). The main pathway that sustains this excessive pro-fibrotic response is the TGFβ pathway, which is overactivated in DMD (*15*, *16*).

In patients with DMD, TGFβ levels, monitored by the nuclear accumulation of phosphorylated SMAD2/3 (pSMAD2/3) transcriptional modulators, are elevated in both plasma and muscle and were shown to contribute to pathological fibrosis (*17*). However, the signaling pathways contributing to human DMD fibrosis, and the signals coordinating diverse cell types to maintain or restore muscle tissue homeostasis, remain largely unknown. Yet, TGFβ pathway overactivation is the common feature observed in dystrophin-deficient myotubes in vitro independently of the type of mutation (*15*, *16*), and exacerbated TGFβ/SMAD pathway has been shown to lead to fusion defects in in vitro DMD myotubes. Moreover, the TGFβ pathway has been described as a molecular brake of myoblast fusion and, thus, muscle regeneration, while its inhibition leads to the formation of giant myofibers in vivo (*18*).

SETDB1 has been shown to regulate the TGFβ response in cancer and in T cells (*19*–*22*) and pulmonary fibrosis (*23*) contexts. Here, we investigated the SETDB1/TGFβ interplay in a model of DMD myotubes derived from hiPSCs or immortalized myoblasts (*15*, *24*).

We show that SETDB1 contributes to the deregulated TGFβ signaling in DMD, indicating that SETDB1 may participate in muscle dysfunction and increased fibrosis. TGFβ induces nuclear accumulation of SETDB1 in healthy myotubes, while SETDB1 is constantly accumulated in DMD myotube nuclei with intrinsic overactivated TGFβ pathway. Moreover, transcriptomics showed that SETDB1 silencing attenuates the TGFβ–dependent pro-fibrotic and anti-differentiation response in DMD myotubes. Many TGFβ/SETDB1-dependent genes code for secreted proteins involved in ECM remodeling and inflammation. Conditioned medium (CM) assays show that TGFβ–treated myotubes with SETDB1 LOF produce a secretome that reduces the negative impact of TGFβ response on myoblast differentiation and impairs myoblast pro-fibrotic response. Our findings point to a role of SETDB1 in the fine-tuning of the TGFβ response in muscles, which is deregulated in DMD, participating in fibrosis.

## RESULTS

### SETDB1 relocalizes in the nuclei of healthy differentiated human myotubes in response to TGFβ pathway activation

All our experiments were conducted in human models generated from either human-induced pluripotent stem cells [hiPSCs; which we have already described (*15*), or from immortalized myoblasts derived from healthy individuals or patients with DMD (*24*)]. hiPSCs can be directly induced to differentiate into myotubes using a transgene-based epigenetic reprogramming strategy (*15*).

**Fig. 1.**
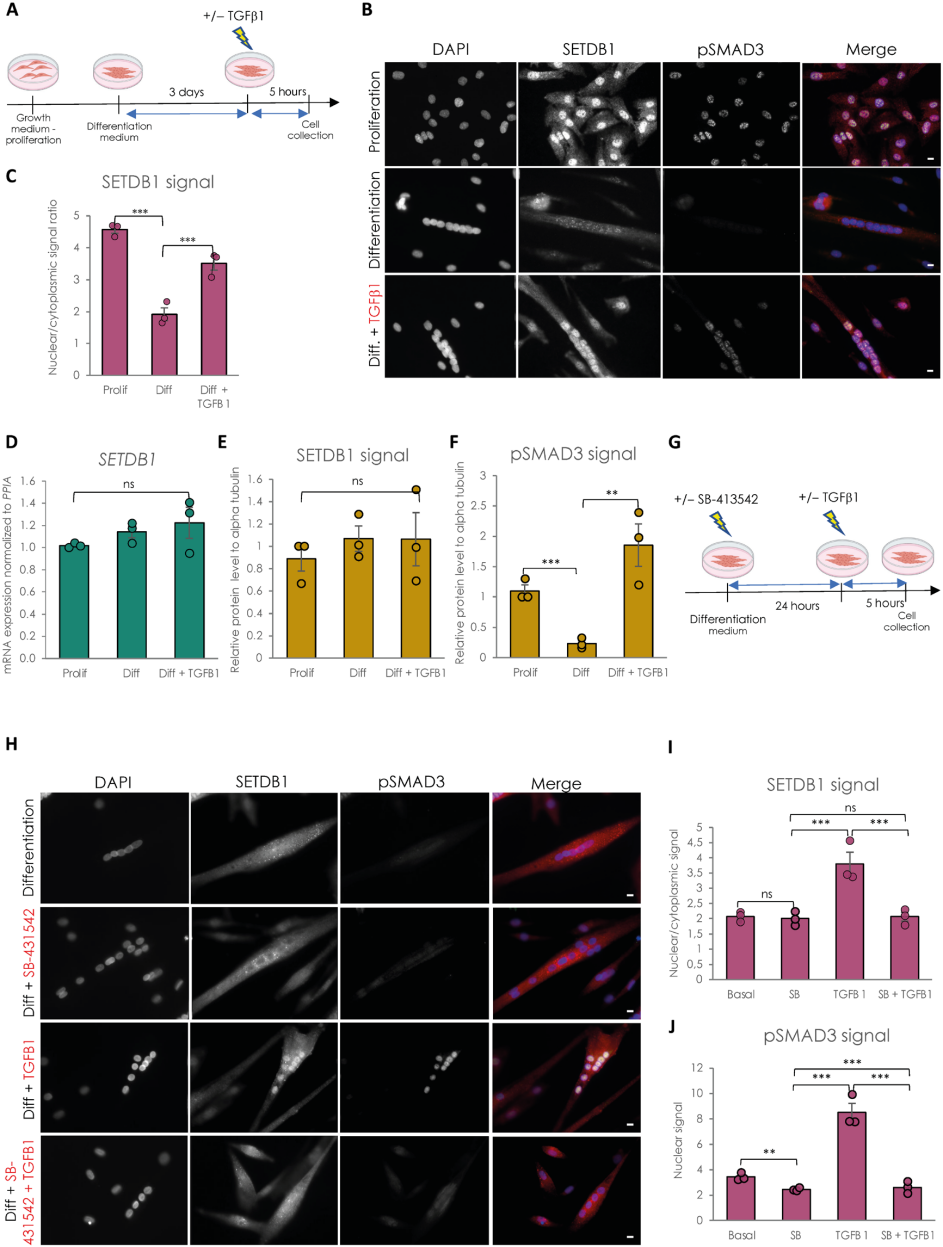
SETDB1 is excluded from myotube nuclei upon differentiation and can be relocated in response to TGFβ pathway activation. (**A**) Scheme of experimental design. Cells were collected in proliferating phase (myoblasts) or after 3 days of differentiation (myotubes) with or without TGFβ1 treatment at 20 ng/ml. (**B**) Immunostaining of SETDB1 (red) and phospho-SMAD3 (green). Nuclei were stained with 4′,6-diamidino-2-phenylindole (DAPI) (blue). Scale bars, 10 μm. (**C**) Quantification of SETDB1 nuclear/cytoplasmic signal ratio. (**D**) Quantitative reverse transcription polymerase chain reaction (RT-qPCR) of *SETDB1* in healthy proliferating myoblasts and differentiated myotubes +/− TGFβ1. (**E** and **F**) Quantification of SETDB1 and phospho-SMAD3 protein levels measured by Western blot (see fig. S1C) in proliferating myoblasts and myotubes treated or not with TGFβ1 in total protein extracts. Alpha tubulin was used as a loading control to normalize samples. (**G**) Scheme of experimental design with TGFβ inhibitor SB-431542. (**H**) Immunostaining of SETDB1 (red) and phospho-SMAD3 (green). Nuclei were stained with DAPI (blue). SB-431542 blocks SETDB1 relocalization in myotube nuclei upon TGFβ1 treatment. Scale bars, 10 μm. (**I** and **J**) Quantification of SETDB1 nuclear/cytoplasmic signal ratio (I) and phospho-SMAD3 nuclear signal (J). For all panels, statistics were performed on ≥3 biological replicates (>100 nuclei for immunostaining quantification), and data are represented as average ± SEM. **P < 0.01; ***P < 0.001 (unpaired Student’s *t* test). ns, not significant.

We have already shown that in murine myoblasts Setdb1 is both cytoplasmic and nuclear, and during terminal muscular differentiation, Setdb1 is exported to the cytoplasm in a Wnt-dependent manner, allowing the derepression of muscle genes (*4*). Thus, we have first investigated, and quantified (as depicted in fig. S1A), the subcellular localization of SETDB1 in proliferating versus differentiating healthy human myoblasts and in myotubes in response to TGFβ/SMAD pathway activation (Fig. 1A). TGFβ/SMAD pathway is constitutively activated in human proliferating myoblasts, while nuclear phospho-SMAD3 (p-SMAD3) levels decrease upon differentiation (Fig. 1B and fig. S1B), as already shown in murine muscle cells (*18*). Differentiated myotubes are still responsive to TGFβ/SMAD pathway activation since p-SMAD3 nuclear levels are restored upon TGFβ1 treatment (Fig. 1F and fig. S1B). TGFβ treatment efficiency was evidenced by the activation of many TGFβ target genes, including the *TGF*β gene itself, concomitant to a decrease in many differentiation genes (fig. S1D).

We have confirmed in human muscle cells that SETDB1 is also highly enriched in the nuclei of proliferating myoblasts while it is depleted in nuclei of differentiated myotubes (Fig. 1, B and C), as in murine cells (*4*). TGFβ/SMAD pathway activation in muscle cells is correlated with SETDB1 presence in nuclei (Fig. 1, B and C) and we were able to trigger SETDB1 nuclear enrichment in differentiated myotubes after TGFβ1 treatment (Fig. 1, B and C), in the absence of significant changes in SETDB1 mRNA (Fig. 1D) and protein levels (Fig. 1E and fig. S1C), neither during terminal differentiation nor in response to 24-hour TGFβ1 treatment. This result was confirmed in our healthy human hiPSC-derived model (*15*) of muscle cells (fig. S2C, top). We next used the ALK4/5/7 inhibitor SB-431542 in myotubes to block TGFβ pathway activation before TGFβ1 addition (Fig. 1G). The SB-431542 efficiency was evidenced by its effect on phospho-SMAD3 nuclear levels and TGFβ target genes and *MYOD1* expression (Fig. 1, H and J, and fig. S1E). Autocrine TGFβ signal blocking does not change SETDB1 localization in myotubes in basal condition but blocks SETDB1 nuclear relocalization in healthy myotubes in response to TGFβ1 (Fig. 1, H and I).

Together, these data showed that SETDB1 is excluded from the nucleus upon terminal differentiation of human myoblasts, and TGFβ pathway activation is directly responsible for SETDB1 nuclear relocalization in myotube nuclei.

### SETDB1 is accumulated in DMD myotube nuclei in a TGFβ–dependent manner

We next investigated SETDB1 level and subcellular localization in the context of DMD in which the TGFβ pathway is overactivated both *in vivo* (*17*, *25*) and in *in vitro* cellular models (*15*, *16*).

**Fig. 2.**
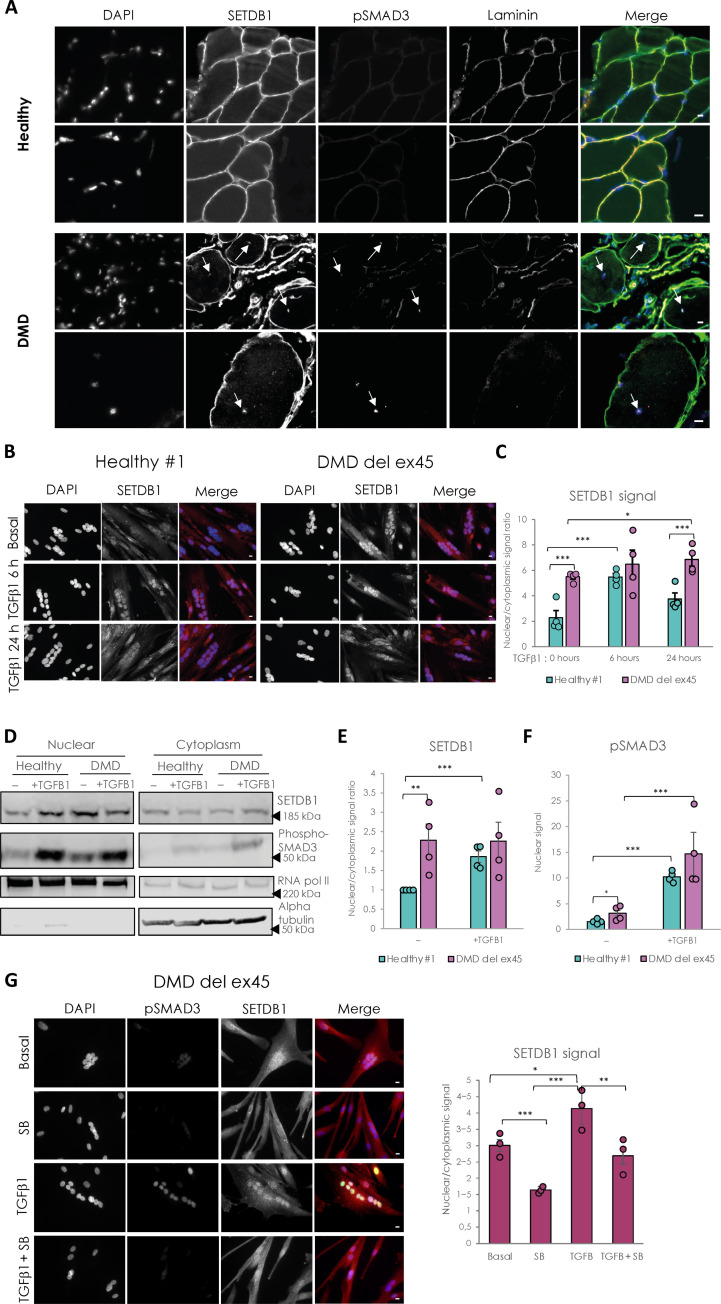
DMD-differentiated muscle cells display constitutive relocalization of SETDB1 in the nuclei and higher activation of the TGFβ/SMAD pathway. (**A**) Immunostaining of SETDB1 (green), pSMAD3 (magenta), and laminin (red) on histological slides from healthy individual or DMD patient paravertebral muscles. Nuclei were stained with DAPI (blue). White arrows identify centrally localized nuclei of damaged myofibers in the DMD muscle section. Scale bars, 10 μm. (**B**) Immunostaining of SETDB1 (red) in healthy #1 and DMD del ex45 myotubes. Nuclei were stained with DAPI (blue). Scale bars, 10 μm. (**C**) Quantification of SETDB1 nuclear/cytoplasmic signal ratio. (**D**) Western blot of nuclear and cytoplasmic fractions of healthy and DMD myotubes in response to TGFβ1 showing protein levels of SETDB1 and phospho-SMAD3. RNA polymerase II and alpha tubulin were used as loading controls of nuclear and cytoplasmic fractions, respectively. (**E** and **F**) Quantification of SETDB1 nuclear/cytoplasmic ratio (E) and phospho-SMAD3 nuclear signal (F). (**G**) Immunostaining of SETDB1 (red) and phospho-SMAD3 (green) in DMD del ex45 myotubes and quantification of SETDB1 nuclear/cytoplasmic signal ratio. Nuclei were stained with DAPI (blue). Scale bars, 10 μm. For all panels, statistics were performed on ≥3 biological replicates (>100 nuclei for immunostaining quantification), and data are represented as average ± SEM. *P < 0.05; **P < 0.01; ***P < 0.001 (unpaired Student’s *t* test).

First, immunohistochemistry assays show that SETDB1 co-localizes with phospho-SMAD3 in the centrally localized nuclei in DMD patient muscle histological sections (Fig. 2A). To further confirm this observation more quantitatively, we efficiently differentiated three healthy myoblasts or hiPSC lines and three DMD lines carrying different *Dystrophin* mutations (myoblasts with exons 45 to 52 deletion (Fig. 2B), myoblasts with point mutation in exon 59 (fig. S2B), and hiPSCs with exon 45 deletion (fig. S2C). Compared to healthy differentiated myotubes, DMD myotubes display overactivated TGFβ/SMAD pathway, as depicted by *TGF*β1 mRNA expression (fig. S2A) and nuclear phospho-SMAD3 protein level (Fig. 2, D and F) (*15*, *16*), as expected.

The quantification of SETDB1 nuclear/cytoplasmic ratio in healthy myotubes shows that SETDB1 translocated in the nuclei after 6 h of TGFβ1 treatment and this relocalization tends to decrease after 24 h (Fig. 2, B and C, and fig. S2B). In DMD myotubes, SETDB1 is accumulated in the nuclei at a steady state and is persistent in response to TGFβ pathway activation (DMD exon 45 deletion in myoblasts or hiPSCs, Fig. 2, B and C; DMD point mutation, fig. S2B) and is associated with a higher activation of *TGF*β1 expression (fig. S2A). The effect of TGFβ pathway activation on the subcellular localization of SETDB1 is further confirmed by the western blotting of nuclear and cytoplasmic fractions of healthy and DMD myotubes (Fig. 2, D to F) and also in our hiPSC-derived DMD myotubes (exon 45 deletion; fig. S2C). We also blocked the autocrine signal of TGFβ in DMD myotubes with SB-431542 and found that it leads to decreased SETDB1 levels in nuclei with and without TGFβ1 treatment (Fig. 2G). Overall, these data showed an effect of TGFβ on SETDB1 nuclear relocalization in at least three different DMD patient myotubes with different *Dystrophin* gene mutations.

To elucidate the molecular mechanisms underlying SETDB1 relocalization in DMD myotubes, we first tested if SETDB1 nuclear translocation in response to TGFβ was dependent on SMAD3, which is the effector translocating to the nucleus in response to TGFβ. Our results show that SETDB1 nuclear enrichment in response to TGFβ is not dependent on the presence of SMAD3 protein (fig. S2, D to F).

SETDB1 is known to bear numerous posttranslational modifications including many phosphorylations (www.phosphosite.org/proteinAction.action?id=9498&showAllSites=true). We thus hypothesized that the phosphorylation status of SETDB1 might correlate with its subcellular localization. To check this, we tested the migration profile of SETDB1 in several subcellular compartments. Our data showed that SETDB1 from the cytosolic and peri-nuclear fractions migrates higher compared to nuclear SETDB1 (fig. S2G), indicating possible hyperphosphorylation in these compartments. However, these observations require further investigation.

Together, these data showed that TGFβ induces SETDB1 nuclear enrichment in normal and DMD myotubes, regardless of the genetic background. This suggested that SETDB1 could modulate the cellular response to TGFβ.

### SETDB1 loss of function attenuates the TGFβ response in human DMD myotubes

**Fig. 3.**
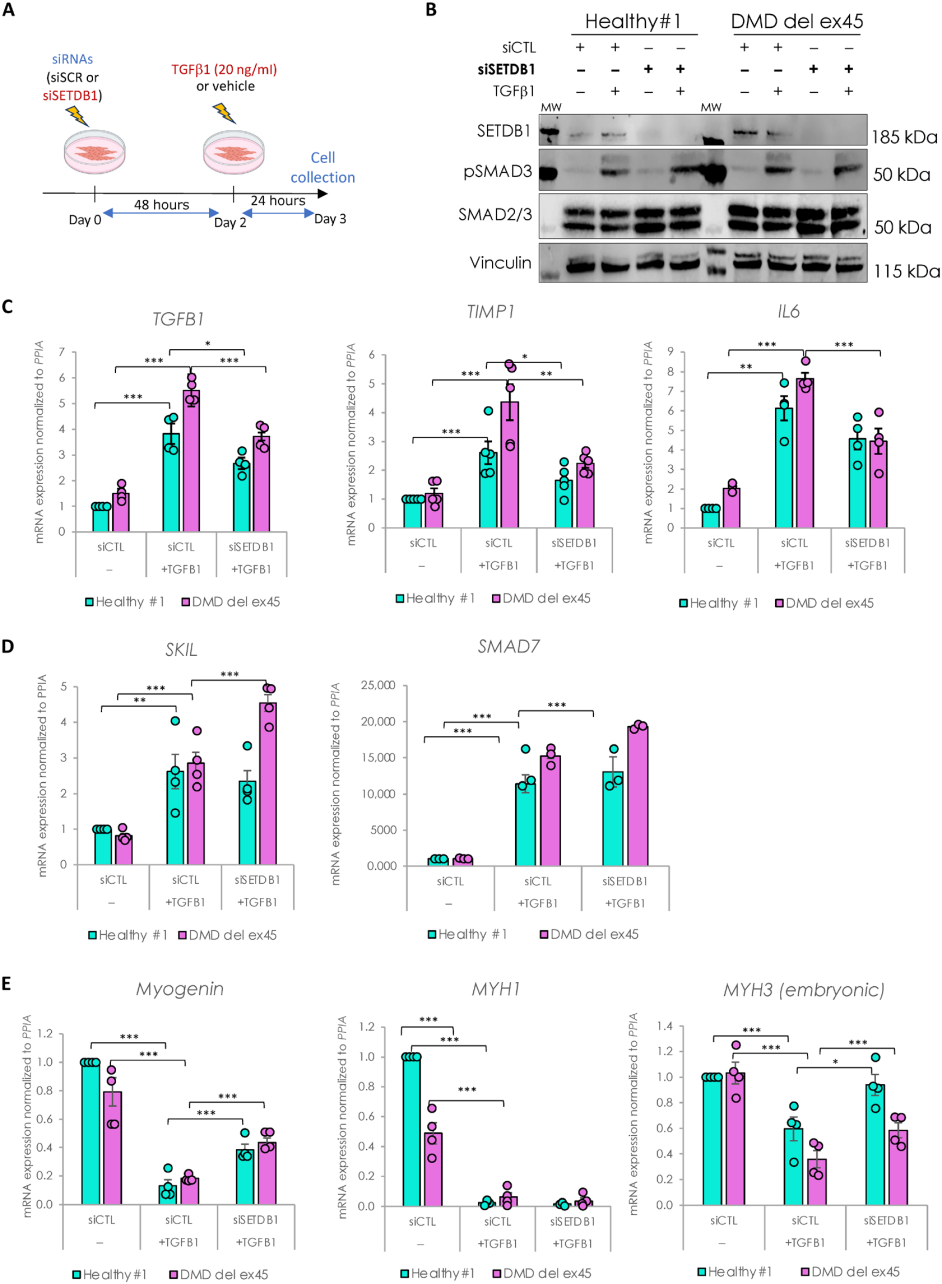
SETDB1 silencing leads to a decreased response to TGFβ1 in DMD myotubes while key myogenic markers increase. (**A**) Scheme of experimental design. Cells were treated with siRNAs scrambled (siSCR) or against SETDB1 for 2 days after 3 days of differentiation (myotubes) and then treated or not with TGFβ1 at 20 ng/ml. (**B**) Western blot showing SETDB1, phospho-SMAD3, and total SMAD2/3 protein levels. Vinculin was used as a loading control. siCTL, scrambled siRNA (**C**) RT-qPCR of TGFβ/SMAD pathway known targets *TGF*β1, *IL6*, and *TIMP1* in healthy and DMD myotubes +/− siSETDB1 +/− TGFβ1. (**D**) RT-qPCR of TGFβ/SMAD pathway inhibitors *SKIL* and *SMAD7* in healthy and DMD myotubes +/− siSETDB1 +/− TGFβ1. (**E**) RT-qPCR of myogenic markers *Myogenin*, *MYH1*, and *MYH3 (embryonic MHC).* For all panels, statistics were performed on ≥3 biological replicates, and data are represented as average ± SEM. *P < 0.05; **P < 0.01; ***P < 0.001 (unpaired Student’s *t* test).

To investigate the role of SETDB1 in the TGFβ response, we performed SETDB1 loss of function (LOF) in both healthy and DMD myotubes treated or not with TGFβ1 (as depicted in Fig. 3A). While the small interfering RNA (siRNA)–mediated SETDB1 knockdown (KD; more than 80%) was efficient (Fig. 3B and fig. S3A), it did not show any effect on the levels of SMAD2/3 proteins nor their phosphorylation levels (Fig. 3B). However, SETDB1 LOF led to a decrease in the TGFβ response, as depicted by the expression of ad hoc TGFβ target genes involved in pro-fibrotic response and inflammation, such as *TGF*β1, *TIMP1* (*tissue inhibitor of metalloproteinases 1*), *IL6* (*interleukin-6*), *FN1* (*fibronectin 1*), and *CTGF* (*connective tissue growth factor*) (Fig. 3C and fig. S3C). Note that SETDB1 KD alone, in the absence of TGFβ, does not affect or only slightly the expression of these genes (fig. S3, D and E). Since these TGFβ target genes are down-regulated in SETDB1 KD condition, we hypothesized that in normal conditions SETDB1 could target and repress inhibitors of the TGFβ pathway. A gene candidate approach showed that SETDB1 LOF led to an increase in *SKIL* and *SMAD7* genes only in DMD (Fig. 3D and fig. S3B), two known inhibitors and fine tuners of the TGFβ pathway, while in the absence of TGFβ, SETDB1 KD alone does not affect or only slightly the expression of these genes (fig. S3, D and F). Of note, concomitant to the attenuation of the TGFβ response, SETDB1 LOF in 3-day differentiated myotubes induced a significant increase in muscle early differentiation marker *Myogenin* and regeneration marker *MYH3 (embryonic myosin heavy chain*), but not in the muscle late differentiation marker *MYH1* (Fig. 3E and fig. S3B). Of note, SETDB1 KD alone also showed an effect on the expression of muscle differentiation genes (Fig. 3E and fig. S3G).

Thus, SETDB1 LOF attenuates the TGFβ1–induced fibrotic response while promoting regeneration and could be beneficial in patients with DMD.

### SETDB1 alters the TGFβ–regulated secretome in human DMD myotubes

To deepen the role of SETDB1 in the TGFβ response at the global level, we performed RNA sequencing comparing DMD myotubes with healthy myotubes in response to TGFβ, with or without SETDB1 LOF. Principal components analysis of gene expression levels showed that the triplicates and duplicates were distinctively separated and that the main source of variability between samples is the cell line source, healthy versus DMD. The second source seems to be the effect of the TGFβ treatment and SETDB1 silencing (fig. S4A).

**Fig. 4.**
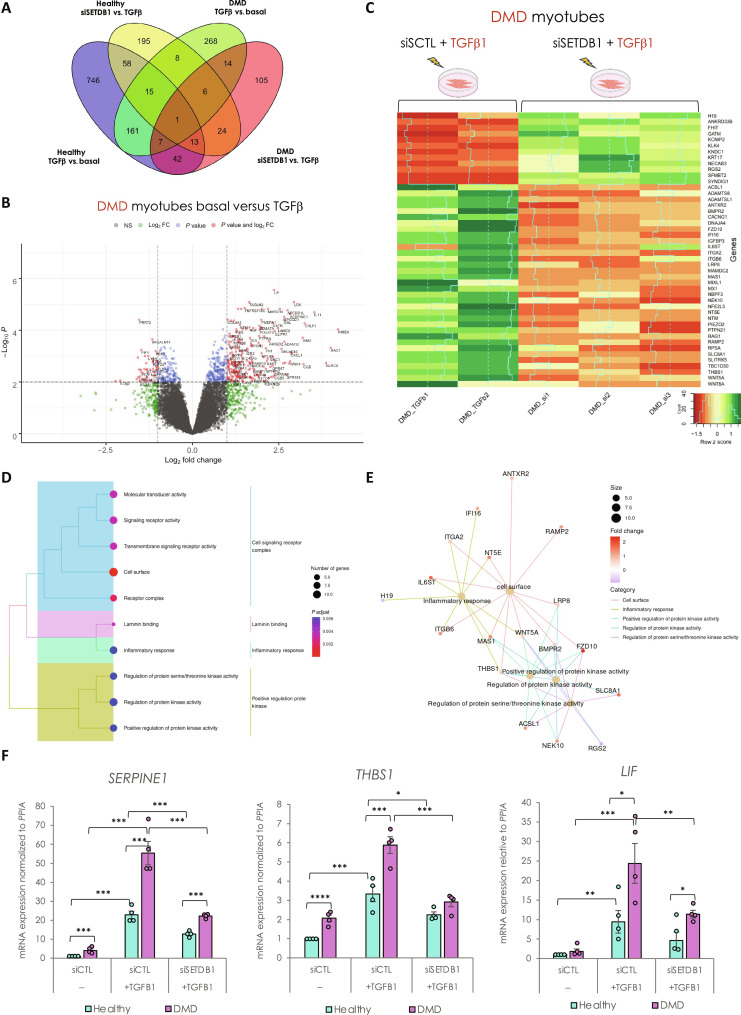
SETDB1 silencing in DMD myotubes leads to a decreased expression of TGFβ–dependent genes involved in ECM remodeling, receptor signaling transduction, and inflammation. (**A**) Venn diagram shows that a different set of TGFβ1–responsive genes were differentially expressed upon SETDB1 silencing in healthy and DMD myotubes. (**B**) Volcano plot of differentially expressed genes in DMD myotubes +/− TGFβ1. (**C**) Heatmap of expression level *z*-scores for DEGs in DMD myotubes for TGF-β versus TGFβ + siSETDB1 comparison. Some genes found to be increased upon TGFβ1 treatment decrease upon SETDB1 silencing (*IGFBP3*, *THBS1*, and *IL6ST*). (**D** and **E**) Enriched categories tree plot (D) and enriched gene-concept network for biological pathways deregulated for TGFβ versus TGFβ + siSETDB1 in DMD myotubes. (**F**) mRNA levels of gene validated by RT-qPCR involved in ECM remodeling (*THBS1* and *SERPINE1*) and inflammation (*LIF*). For all panels, statistics were performed on ≥3 biological replicates, and data are represented as average ± SEM. *P < 0.05; **P < 0.01; ***P < 0.001 (unpaired Student’s *t* test).

To track changes in gene expression, first, in response to TGFβ, we performed differential gene expression analysis (Fig. 4A). We identified 1043 TGFβ–dependent differentially expressed genes (DEGs) in healthy myotubes (489 up- and 554 down-regulated genes) and only 480 in DMD myotubes (363 up- and 117 down-regulated genes) (top genes in tables S1 and S2). These data showed that while the TGFβ pathway is already intrinsically activated in DMD myotubes, it is not saturated and can still be further activated *in vitro* (see also Figs. 2 and 3) (*15*). Only 184 TGFβ–dependent genes (corresponding to 18% of healthy and 39% of DMD DEGs) are commonly deregulated in healthy and DMD conditions (Fig. 4A).

Gene Ontology (GO) analysis of the TGFβ–responsive genes in healthy myotubes showed substantial enrichment in terms related to the regulation of MAPK cascade, negative regulation of locomotion, cellular component movement, cell adhesion, SMAD protein phosphorylation, migration, motility, chemotaxis, vessel, and nervous development (fig. S4B). While in DMD myotubes, GO showed that the main categories are related to cytokine-mediated signaling, ECM remodeling and organization, inflammation, SMAD protein phosphorylation, and positive regulation of cell motility (Fig. 4B and fig. S4C), reminiscent of disease traits. Collectively, these data validated the responsiveness of both healthy and DMD myotubes to the TGFβ treatment.

Then, we checked the DEGs in SETDB1 LOF condition, both in healthy and DMD myotubes treated with TGFβ. We found that SETDB1 LOF affected 320 genes in response to TGFβ in healthy myotubes (Fig. 4A and top genes in table S3). To determine the signature of the transcriptional changes, we applied gene set enrichment analysis (GSEA) and found that the SETDB1-dependent genes belong to ECM and cell surface categories (fig. S4D). Together, these data already suggest that SETDB1 LOF affects genes coding for secreted factors.

In DMD myotubes, SETDB1 LOF affected 212 genes in response to TGFβ (Fig. 4, A and C, and top genes in table S4), 120 were less abundant and 92 more abundant. In particular, SETDB1 LOF induced less abundant mRNAs associated with ECM remodeling, inflammation, and fibrosis, including *THBS1* (*thrombospondin-1*), *Myostatin*, *BMPR2 (bone morphogenetic protein receptor type 2)*, *ADAMTS8 (ADAM metallopeptidase with thrombospondin type 1 motif 8)*, *MMP14 (matrix metallopeptidase 14)*, *LIF (leukemia inhibitory factor)*, *SERPINE1 (plasminogen activator inhibitor type 1 or PAI1)*, *WNT5A*, and *MAMDC2 (MAM domain containing 2)* (Fig. 4, C and F, and fig. S4E). All these genes, gene categories, and pathways are notably involved in the DMD disease traits. Furthermore, enrichment analysis for biological processes and gene network of biological pathway analyses highlighted that the SETDB1-dependent genetic programs in DMD myotubes in response to TGFβ are mainly involved in inflammation, signaling, and cell surface (Fig. 4, D and E). SETDB1 KD alone, in the absence of TGFβ, affects to a lesser extent the expression of the tested genes (fig. S3, D and H).

To evaluate the correlation between chromatin remodeling and gene expression following SETDB1 LOF in myotubes, we tried to conduct ATAC-seq assays. However, the process proved more challenging than anticipated, particularly during the tagmentation step. While samples from healthy myotubes successfully passed quality control, those from DMD myotubes did not (fig. S4F), suggesting that the enrichment of SETDB1 in the nucleus in DMD myotubes might affect chromatin accessibility.

Next, we wanted to test whether the SETDB1-mediated TGFβ response in DMD myotubes is dependent on SETDB1 enzymatic activity. To address this, we designed rescue experiments involving the expression of exogenous SETDB1 variants, either WT or catalytically-dead mutant forms, while simultaneously knocking down endogenous SETDB1 using an siRNA targeting the 3′ untranslated region of its mRNA. For this purpose, we first used lentiviral infection to establish DMD myoblast cell lines expressing either wild-type (WT) SETDB1 or a catalytic-dead mutant (H1224K point mutant), under doxycycline-dependent control (fig. S5). However, the expression of exogenous proteins failed to replicate the endogenous scenario. Moreover, the protein levels of SETDB1 catalytic-dead mutant were notably lower than that of WT SETDB1 upon doxycycline induction in all tested conditions making it challenging to compare the effects (fig. S5A). Our data revealed an unexpected phenotype in myotubes expressing the catalytic-dead SETDB1. In contrast to controls or those expressing WT SETDB1, the myotubes with catalytic-dead SETDB1 displayed a less organized structure and a higher fusion index (fig. S5, B to G). This posed a challenge in performing rescue assays designed to address the question of the necessity of SETDB1 catalytic activity in DMD myotubes. Notably, the SETDB1 catalytic-dead mutant induced this distinct phenotype even at very low expression levels (without doxycycline; fig. S5, F and G) and in the presence of the endogenous SETDB1. Therefore, caution is warranted in interpreting the implications of our findings, as the unexpected effects associated with catalytic-dead SETDB1 expression may introduce additional layers of complexity to the observed outcomes.

Together, here, we validated the efficacy of TGFβ to induce fibrotic and inflammation genetic programs and highlighted that SETDB1 silencing alters the transcription of many secreted factors (secretome) and ECM components, attenuating the deleterious effect of TGFβ overactivation in DMD myotubes.

### SETDB1 LOF in myotubes has a beneficial impact on the TGFβ−induced secretome: It improves muscle differentiation and reduces fibrosis

**Fig. 5.**
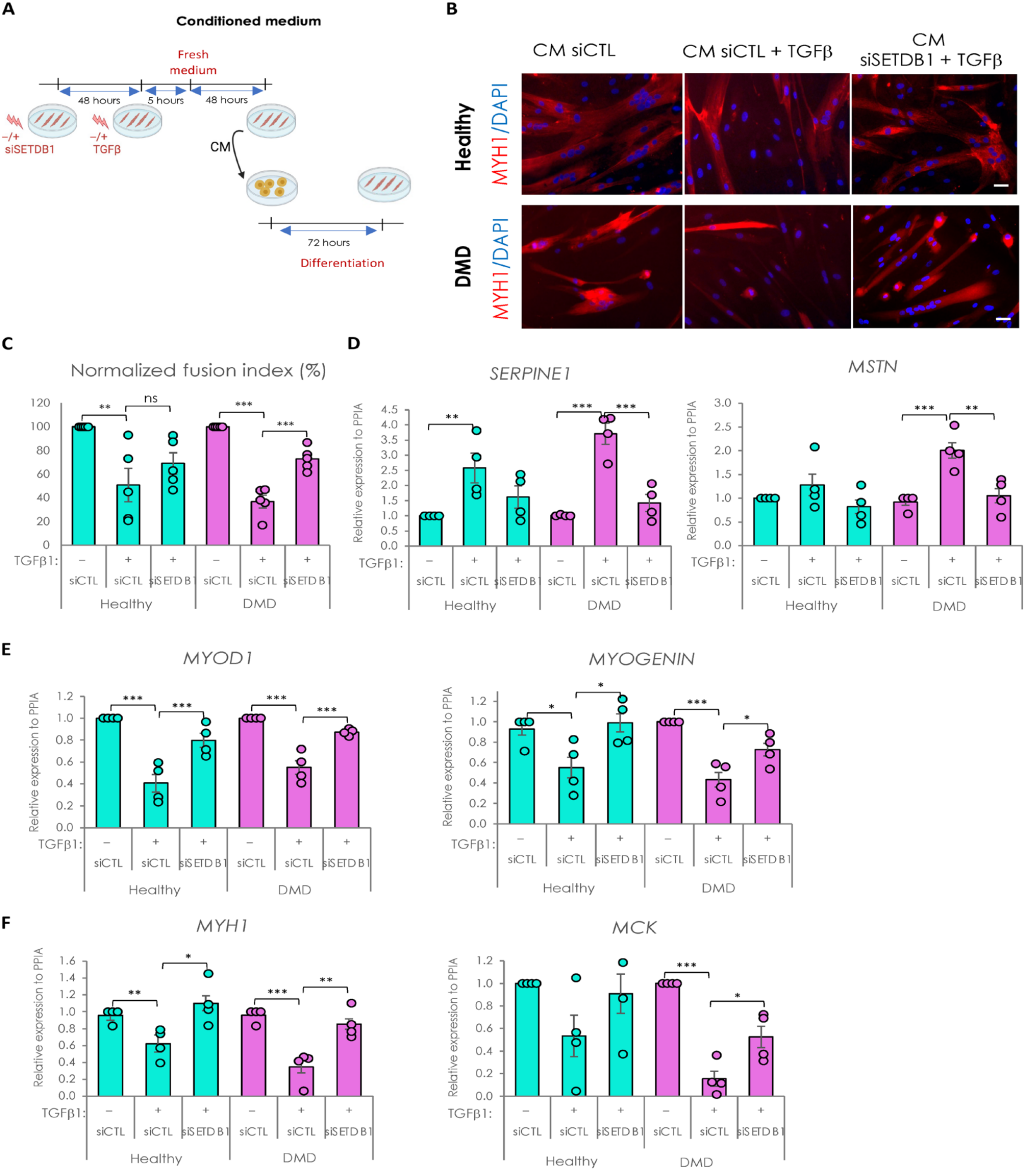
Secretome of SETDB1 deficient myotubes in response to TGFβ1 reduces the negative impact of TGFβ treatment on myoblast differentiation. (**A**) Diagram of the conditioned medium experiments. (**B**) Immunofluorescence of myosin heavy chain (red) and nuclei staining with DAPI (blue). Scale bars, 50 μm. (**C**) Quantification of fusion index after 6 days of differentiation in the conditioned medium. (**D**) RT-qPCR of fibrotic markers *SERPINE1* and *MSTN* after 3 days in the conditioned medium. (**E**) RT-qPCR of early myogenic markers *MYOD1* and *MYOGENIN*. (**F**) RT-qPCR of late myogenic markers *MYH1* and *MCK.* For all panels, statistics were performed on ≥3 biological replicates (>100 nuclei for immunostaining quantification), and data are represented as average ± SEM. *P < 0.05; **P < 0.01; ***P < 0.001 (unpaired Student’s *t* test).

So far, our data showed that SETDB1 LOF in myotubes could potentially modulate the impact of TGFβ on surrounding cells and influence the process of muscle regeneration. To test this, we produced CM in healthy or DMD myotubes with or without SETDB1 LOF, treated or not with TGFβ (Fig. 5A). Then, CM from healthy myotubes was applied on confluent healthy myoblasts, and CM from DMD myotubes was applied on DMD myoblasts (Fig. 5A).

As a control, we first checked whether CM could affect myoblast differentiation as compared to fresh differentiation medium and found no changes (fig. S6, A to C). Thus, receiving myoblasts treated with fresh medium or CM supplemented with TGFβ1 showed a less efficient differentiation (fig. S6, A to C). In the absence of medium change, CM from TGFβ–treated myotubes completed impaired fusion, as expected, meaning that TGFβ1 is not consumed by the cells and remains high in the CM and hides the myotube response (*18*).

We then included medium change after acute TGFβ1 treatment to study the intrinsic myotube pro-fibrotic response (Fig. 5A). Our results showed that DMD myoblasts, but not healthy ones, have a decreased fusion index when differentiated with CM from TGFβ1–treated myotubes, but myoblasts incubated with CM from TGFβ1–treated and SETDB1 LOF myotubes show a more normal fusion index (Fig. 5, B and C). SETDB1 KD alone in myotubes, in the absence of TGFβ, does not affect the fusion index of the myoblasts receiving the CM (fig. S6E).

The discrepancy between the effects of the CM in healthy versus DMD myoblasts could be due to the higher differentiation potential of the healthy myoblasts (fig. S6D).

The receiving myoblasts incubated with CM from TGFβ–treated and SETDB1 LOF myotubes showed a significant decrease in fibrotic markers *SERPINE1* and *MSTN* (*myostatin*) (Fig. 5D). Concomitantly, they displayed an increase in differentiation markers expression such as *MYOD1*, *Myogenin*, *MCK* (*muscle creatine kinase*), and *MYH1* (Fig. 5, E and F). SETDB1 KD alone in myotubes, in the absence of TGFβ, does not affect the expression of muscle differentiation genes in the myoblasts receiving the CM (fig. S6F).

Together, these data show that the TGFβ pathway activation could be attenuated by targeting SETDB1 in patients with DMD. Thus, SETDB1 LOF in myofibers could be beneficial for the myofibers themselves but also to the differentiation of the surrounding myoblasts and potentially limit fibrosis.

## DISCUSSION

Tissue repair, such as muscle regeneration after injury, involves many cell types that communicate together through secreted molecules, the so-called secretome, to orchestrate the replacement of damaged myofibers with new functional ones and re-establish tissue homeostasis. Thus, in addition to their contractile properties, myofibers have also a central role in cell-cell communication since they are able to secrete the so-called myokines, especially during regeneration and differentiation, including dedicated vesicles acting on muscle adaptation to damage or exercise (*26*). This myofibers secretome plays important roles in intercellular communication of the muscle resident cells, i.e., MuSCs, myofibers themselves, the fibro-adipogenic progenitors (FAPs), and macrophages (*27*).

Although FAPs and macrophages have been described as major TGFβ sources, and as fibrosis and inflammation effectors, myofibers are also responsive to TGFβ1 and have been shown to participate in TGFβ/SMAD response during muscle regeneration in vivo (*28*, *29*). TGFβ–induced fibrosis is a key pathological feature in muscle diseases, such as DMD, and TGFβ overactivation worsens muscle degeneration by preventing proper repair. Moreover, although myoblasts display a TGFβ/SMAD autocrine signal required for proliferation, the TGFβ pathway prevents myoblast fusion by controlling actin remodeling and its activation decreases upon muscle differentiation. DMD muscle cellular models have been shown to recapitulate TGFβ/SMAD overactivation pathological features *in vitro* associated with muscle defects and fibrosi*s* (*15*, *16*). Hence, targeting TGFβ–induced pro-fibrotic response in DMD to slow down disease progression appears as a promising therapeutic approach.

TGFβ signaling nuclear endpoint mediated by the two major TGFβ downstream transcription factors SMAD2 and SMAD3 also involves many chromatin-modifying enzymes including SETDB1.

SETDB1 regulates different cell fates including stemness and terminal differentiation (*2*). SETDB1 is also involved in fibrosis (*23*), inflammatory response, and diseases (*5*, *30*–*32*) and has been linked to TGFβ response in many non-muscle contexts (*19*–*23*). SETDB1 has been previously described by us and others as a major negative regulator of muscle terminal differentiation through repression of muscle gene expression in myoblasts nuclei (*4*, *33*). SETDB1 is responsive to Wnt signaling that is known to have a pro-differentiation effect on myoblasts and causes SETDB1 export to the cytoplasm allowing muscle gene derepression. SETDB1 has also been shown to be a regulator of the TGFβ/SMAD pathway in cancer (*19*–*22*) and pulmonary fibrosis (*23*) contexts. In this study, we have shown that SETDB1 nuclear localization follows TGFβ/SMAD activation in human muscle cells. SETDB1 is exported to the cytoplasm during muscle terminal differentiation, while TGFβ/SMAD pathway activation decreases, but it can be transiently relocalized in myotube nuclei in response to TGFβ pathway activation.

In the effort to identify key players of TGFβ response whose dysregulation is a key driver of DMD progression, we hypothesized that SETDB1 could participate in TGFβ response in muscle and, though, could be involved in deregulation of TGFβ pathway in DMD. We found that SETDB1 is constitutively accumulated in DMD myotube nuclei correlating with phospho-SMAD3 high nuclear levels in vivo and in vitro. As expected, DMD myotubes are more responsive to TGFβ activation as compared to healthy ones. We were able to block SETDB1 nuclear localization by inhibiting intrinsic autocrine TGFβ signal in DMD myotubes. We also found that SETDB1 nuclear translocation is not dependent on SMAD3 protein and might involve phosphorylation events. We also performed a LOF of SETDB1 and studied TGFβ–induced pro-fibrotic response. We found that healthy but especially DMD myotubes lacking SETDB1 display an attenuated response to TGFβ/SMAD pathway activation without changing phospho-SMAD3 levels. These results point to an unprecedented link between SETDB1 and TGFβ response in muscle, and more especially in the DMD context. Nonetheless, the exact mechanisms through which SETDB1 localization is controlled by either TGFβ or Wnt pathways are not yet understood.

To better understand how SETDB1 affects TGFβ response in myotubes, we performed a global transcriptomic analysis on healthy and DMD myotubes. We found that SETDB1 LOF leads to a global decrease of RNA levels of the genes coding for factors involved in ECM remodeling, inflammation, and TGFβ and Wnt pathway signaling that respond to TGFβ. Since SETDB1 is known for its repressive action on gene expression, we made the hypothesis that SETDB1 could repress TGFβ inhibitors as already described in the cancer context (*20*). We found that SETDB1 LOF leads to an increased expression of the TGFβ inhibitor genes *SKIL* and *SMAD7*. The precise mechanism through which SETDB1 controls the expression of these genes remains unclear. Chromatin immunoprecipitation assays on SETDB1 and H3K9me3 marks would be required to confirm that SETDB1 represses TGFβ inhibitor expression but remains challenging in myotubes. In general, the mechanisms of action of SETDB1 at the chromatin level are well-documented (*34*, *35*). Therefore, we can speculate that the enrichment of SETDB1 in the nucleus in DMD myotubes might affect not only chromatin accessibility but also 3D genome organization, and these points would be interesting to address in the future.

Last, since most of SETDB1 direct or indirect targets are coding for secreted factors involved in fibrotic response and that TGFβ is known for its anti-differentiation role, we tested the effect of myotube secretome on myoblasts. Moreover, DMD muscle cells display fusion defects in some DMD models of hiPSC-derived myotubes and our model of immortalized myoblast-derived myotubes. Here, TGFβ–treated myotube secretome negatively affects myoblast differentiation and, more specifically, fusion, but SETDB1 LOF in DMD myotubes has a beneficial cell nonautonomous effect on myoblasts fusion. We show here the effect of SETDB1 on myotube secretome that affects environing cells. Targeting SETDB1 in postmitotic myotubes does not seem to have a deleterious effect on cell viability but its targeting in muscle tissue would be challenging since we do not know the impact on other cell types such as FAPs and macrophages. Nevertheless, we highlight interesting, secreted targets displaying aberrant expression in DMD myotubes that could play a role in the deleterious environment of DMD. Moreover, some deregulated targets have an unknown function such as *ANKRD33B* but could be involved in the muscle regeneration process.

In summary, this study highlights the role of SETDB1 in postmitotic muscle cells in adaptation to environmental cues (fig. S7). Although the mechanism(s) involved in SETDB1 regulation and the requirement of its enzymatic activity remain elusive, we unraveled a functional effect of its LOF on muscle cell differentiation and shed light on gene networks under SETDB1 control that would be worthy of study individually in the context of DMD. At last, we did not investigate Wnt and TGFβ interaction in this study but it seems that SETDB1 acts as a mediator of this pathway communication in muscle. Although Wnt and TGFβ are described as having opposite effects on muscle terminal differentiation, some studies also described their collaboration in muscle (*36*, *37*). Here, we showed in vitro the cell nonautonomous effect of SETDB1 inhibition in myotubes on the surrounding myoblasts differentiation through the control of secreted factor expression induced by the TGFβ/SMAD pathway. Nevertheless, it is unclear if this effect could be beneficial in vivo during regeneration since some of the targets have been described in the literature for their double-edged effect on muscle regeneration and this could depend on the cell type involved. A fine-tuned balance has to be established in muscle tissue to allow a proper regeneration and SETDB1 could be involved in this mechanism by controlling muscle differentiation, but also by regulating myofiber response to environmental cues.

## MATERIALS AND METHODS

### Establishment of stable cell lines and cell culture

The hiPSC lines, control, and DMD, generated from skin fibroblasts from Coriell (Table 1), were obtained from the Marseille Stem Cells platform in Marseille Medical Genetics laboratory and described in (*38*). iPSCs were maintained in culture in mTesR1 medium and dissociated using ReLeSR (STEMCELL Technologies). They were transfected with epB-Puro-TT-mMyoD and epB-Bsd-TT-FlagBaf60c2 by electroporation using the Neon Transfection System as described in (*15*, *16*). The selection was performed using puromycin (5 μg/ml) and blasticidin (10 μg/ml) at the same time.

**Table 1.** Cell line list.

Cells	Reference	Healthy individuals and patients
Induced pluripotent stem cells (iPSCs)	AG08C5 Coriell	Skin fibroblast, 1-year-old healthy male
	GM25313	Skin fibroblast, 13-year-old DMD male patient, deletion ex45
Immortalized myoblasts (hTERT/CDK4) (hMB)	AB1190C16PV	Paravertebral, 16-year-old healthy male
	AB1167C20TFL	Fascia lata, 20-year-old healthy male
	AB1023DMD11Q	Quadriceps, 11-year-old DMD male
	AB1071DMD13PV	Paravertebral, 13-year-old DMD male

Human control and DMD immortalized myoblasts were obtained from AFM-MyoLine. Immortalization was performed as described in (*24*), using human telomerase-expressing and cyclin-dependent kinase 4–expressing vectors. They were cultured on gelatin-coated plates and propagated in Dulbecco’s modified Eagle’s medium (DMEM) high glucose GlutaMAX (Invitrogen, 61965-026)/Medium 199 (Invitrogen, 41150020) 4:1 mixture, supplemented with 20% fetal bovine serum (Sigma-Aldrich, F7524), fetuin (50 μg/ml), insulin (5 μg/ml), basic fibroblast growth factor (0.5 ng/ml), human epidermal growth factor (5 ng/ml), and dexamethasone (0.2 μg/ml; Sigma-Aldrich, D4902).

To generate DMD cell lines expressing either WT or catalytic-dead mutant (HK) SETDB1 forms for the rescue experiments (fig. S5), DMD myoblasts (del 45 to 52) were transduced with lentiviral particles produced in-house. These particles contained a plasmid encoding either Flag-HA-SETDB1-WT or Flag-HA-SETDB1-H1224K [described in (*39*)]. Subsequently, polyclonal populations were selected using blasticidin S HCl (20 μg/ml; Thermo Fisher Scientific, A1113903). The plasmids used in this study are listed in Table 2, and the specified modifications were incorporated through the NEBuilder HiFi DNA Assembly Master Mix (NEB, E2621S). Cloning primers were designed using the NEBuilder Assembly Tool (https://nebuilder.neb.com/). The H1224K mutation was introduced using the Q5 Site-Directed Mutagenesis Kit (NEB, E0552S).

**Table 2.** Plasmid list.

Plasmid	Comment
pCW_TO_SETDB1_WT	Modified from Addgene, #83481, and pREV-FH-SETDB1 plasmids described in (*39*)
pCW_TO_SETDB1_H1224K	Mutagenesis of SETDB1_WT
pMD2.G	Addgene, #12259
psPAX2	Addgene, #12260

### Myogenic differentiation of iPSCs and human-immortalized myoblasts

iPSCs stably expressing MyoD and Baf60C Tet-ON inducible transgenes were propagated in mTeSR1 on Matrigel-coated wells. To induce myogenic differentiation starting from iPSC colonies, doxycycline (200 ng/ml) was added in cells maintained in mTeSR1 (day 0). After 24 hours of treatment with doxycycline (day 1), cells were dissociated as single cells using TrypLE and plated 25.10^3^/cm2 in growth media (GM) [knockout DMEM (Invitrogen) supplemented with 1 mM l-glutamine, 20% knockout serum replacement medium (KOSR, Invitrogen), GlutaMAX 1×, 0.1 mM nonessential amino acids (Invitrogen), streptomycin (50 U/ml; Invitrogen) plus hES cell recovery supplement (10 μM) (Stemgent), and doxycycline]. On day 3, GM medium was switched to differentiation media (DM) [knockout serum-free DMEM containing 1× insulin-transferrin-selenium (Sigma-Aldrich)] supplemented with doxycycline until day 7. Human control and DMD-immortalized myoblasts were grown at >80% confluence, and the medium was switched for DM media.

### Myotube transfection with siRNAs

Three-day-differentiated myotubes cultured in DM were transfected with siRNAs at a final concentration of 70 nM, using the Lipofectamin RNAiMAX transfection agent (Invitrogen, #13778100). Cells were kept for 2 days in a transfection medium before switching to fresh DM. We used the ON-TARGETplus Human SETDB1 siRNA (Dharmacon, L-020070-00-0010); SMAD3 siRNA (Sense: GUGUGAGUUCGCCUUCAAUAU; Antisense: AUAUUGAAGGCGAACUCACAC), and the ON-TARGETplus Non-targeting Control Pool (Dharmacon, D-001810-10) as a scrambled control siRNA.

### Immunofluorescence on cells and tissue

Muscle histological sections were obtained from muscle biopsies of a 15-year-old patient with DMD with paravertebral muscle and a healthy 17-year-old individual. Frozen samples were fixed in ice-cold acetone for 1 min and then air-dried. They were blocked in 4% bovine serum albumin–phosphate-buffered saline (BSA-PBS) solution for 45 min at room temperature (RT), and then incubated with the primary antibodies at 4°C overnight. Secondary antibodies were applied for 45 min at RT in the dark before mounting.

Cells were grown on Matrigel- and gelatin-coated coverslips for iPSCs and human myoblasts, respectively. They were fixed with 4% paraformaldehyde for 20 min, and they were saturated with 50 mM NH4Cl for 10 min at RT. Then, they were permeabilized with 0.5% Triton X-100 for 5 min. Cells were washed three times with PBS between each step. Cells were next incubated with primary antibodies at the concentration indicated in the antibody list below. Alexa Flour 488 or 555 or 647 were used as secondary antibodies (Invitrogen). Nuclei were counterstained with 4′,6-diamidino-2-phenylindole (DAPI).

Images were acquired with a Leica DMI-6000B microscope and analysis was performed using Fiji software. In-house macro was used to perform nuclear and cytoplasmic signal quantification. Acquisition of iPSC-derived myotubes was performed using ImageXpress Micro High Content Screening System. All the conditions were acquired with identical settings and were analyzed with an in-house macro on ImageJ/Fiji to quantify the nuclear and cytoplasmic signal. The used antibodies are listed in Table 3.

**Table 3.** Antibodies list.

Antibody	Reference	Use and dilution
SETDB1 (rabbit)	Santa Cruz, sc-66884	Immunofluorescence (1:200)
SETDB1 (mouse)	Abcam, ab107225	Western blot (1:1000)
SETDB1 (mouse)	Thermo Fisher Scientific, MA5-15722	Immunofluorescence (1:200)
Myosin heavy chain, MYH1E	DSHB	Immunofluorescence (1:10), Western blot (1:200)
Phospho-SMAD3 (rabbit)	Abcam, ab52903	Immunofluorescence (1:200), Western blot (1:1000)
SMAD3 (mouse)	Proteintech, 66516-1-Ig	Western blot (1:1000)
PAI1 (rabbit)	Proteintech, 13801-1-AP	Western blot (1:1000)
RNA polymerase II (rabbit)	Abcam, ab5095	Western blot (1:1000)
Lamin A/C (rabbit)	Proteintech, 10298-1-AP	Western blot (1:1000)
H3 (C-16) (goat)	Santa Cruz, sc-8654	Western blot (1:1000)
Flag M2 (mouse monoclonal)	Sigma-Aldrich, F1804	Western blot (1:1000)

### RNA and quantitative reverse transcription PCR

Total RNA was extracted using RNeasy Micro Kit (Qiagen) following the manufacturer’s procedures. Deoxyribonuclease (Qiagen) treatment was performed to remove residual DNA. One microgram of total RNA was reverse-transcribed with a High-Capacity cDNA Reverse Transcription Kit (Applied Biosystems). Real-time quantitative PCR was performed to analyze relative gene expression levels using SYBR Green Master mix (Applied Biosystems) following the manufacturer’s indications. Relative expression values were normalized to the housekeeping genes mRNA *PPIA* or *UBC*. Primers are listed in Table 4.

**Table 4.** Primer list.

Targets	Forward	Reverse
*SETDB1*	CAATACCGGGACAGTAGCTC	TCTGGTCTTTTGGAGTTCTGC
*TGFB1*	GCCTGAGGCCGACTACTA	CTGTGTGTACTCTGCTTGAACT
*TIMP1*	TTCTGCAATTCCGACCTCG	TCATAACGCTGGTATAAGGTGG
*IL6*	TAGTGAGGAACAAGCCAGAGC	TGGGTCAGGGGTGGTTATTG
*SKIL*	GCCCCAAATGTGTCACTTAC	TCCCACTTTGTTTATGTCTCTGA
*SMAD7*	GTGTTGCTGTGAATCTTACGG	TCGGGTATCTGGAGTAAGGAG
*MYOGENIN*	AATGCAGCTCTCACAGCGCCTC	TCAGCCGTGAGCAGATGATCC
*MYH1*	CTGTTGCAGTTTCTCATTGGTG	CCAGGCAGTACTTCATTGGG
*MYH3 (embryonic)*	CAAGAGTTCTCAGGATGGGAAG	GCCATGTCTTCGATCCTGTC
*FN1*	AGCCGAGGTTTTAACTGCGA	CCCACTCGGTAAGTGTTCCC
*CTGF*	TGTGCACCGCCAAAGAT	GCACGTGCACTGGTACTT
*SERPINE1*	TCCACAAATCAGACGGCAGC	TCGTAGTAATGGCCATCGGG
*THBS1*	CTGGCCCAATGAGAACCTGG	GCCCTGAGTTGGGAAGGTTG
*LIF*	CCAACTGGCACAGCTCAATG	CTTGTCCAGGTTGTTGGGGA
*MSTN*	AGCGATGGCTCTTTGGAAGAT	TTTGGGTTTTCCATCCACTTGC
*BMPR2*	GCAGGTTCTCGTGTCTAGGG	CCTGGTCCCAACAGTCTTCG
*WNT5A*	GCAGCACTGTGGATAACACC	GCTCACCGCGTATGTGAAGG
*ADAMTS8*	GCAGCGCCATGTATCTCACA	TGTGGGGAGGGGCAGG
*ANKRD33B*	GGGGAACACAGCCCTAATCA	GCGTTCCTCCTTTCAAGGTCA
*MMP14*	GGCTGCCTACCGACAAGATT	GCCCTGAGCTCTTCGTTGAA
*MYOD1*	TGCTCCGACGGCATGATGGACTA	TTGTAGTAGGCGCCTTCGTAGCAGTT
*MCK*	TGGAGAAGCTCTCTGTGGAAGCTC	TCCGTCATGCTCTTCAGAGGGTAGTA
*SMAD3*	TGAGGCTGTCTACCAGTTGACC	GTGAGGACCTTGTCAAGCCACT

### Nuclear and cytoplasmic fractionation

Cells were scraped directly in 3 volumes of buffer A [20 mM Hepes (pH 7.0), 0.15 mM EDTA, 0.15 mM EGTA, and 10 mM KCl] supplemented with 0.15 mM spermine, 0.5 mM spermidine, protease inhibitor cocktail 1× (SIGMAFAST), and phosphatase inhibitor cocktail 1× (Sigma-Aldrich). Cells were lysed with 0.5% NP-40 and mixed gently by inversion. Buffer SR (0.88 volume) [50 mM Hepes (pH 7), 0.25 mM EDTA, 10 mM KCl, and 7% sucrose] supplemented with spermine, spermidine, protease, and phosphatase inhibitor cocktails was added before centrifugating the cells for 5 min at 2000*g* at 4°C, and the supernatant was collected as the cytoplasmic fraction. The core nuclei pelleted at 2000*g* were resuspended in 1 volume of low-salt buffer [20 mM tris (pH 7.65), 25% glycerol, 1.5 mM MgCl2, 0.2 mM EDTA, and 20 mM NaCl] with 3× protease and phosphatase inhibitor cocktails. One volume of high-salt buffer (same as low salt but with 900 mM NaCl) supplemented with 5 mM adenosine 5′-triphosphate while vortexing. The nuclei samples were kept on ice for 30 min and mixed by inversion every 5 min. One volume of sucrose buffer [20 mM tris (pH 7.65), 60 mM NaCl, 15 mM KCl, and 0.34 M sucrose] was added before treating the cells with MNase (0.0025 U/μl) for 10 min at 37°C and 1 mM CaCl2. The reaction was stopped with 4 mM EDTA. Samples were next sonicated for 10 min and ultracentrifugated at 40000 rpm at 4°C for 30 min. The supernatant was collected as a nuclear fraction.

### Western blot

Cells were lysed in radioimmunoprecipitation assay buffer [20 mM tris (pH 7.65), 150 mM NaCl, 0.1% SDS, 0.25% NaDoc, and 1% NP-40] supplemented with protease inhibitor 1× (SIGMAFAST) and phosphatase inhibitor 1× (Sigma-Aldrich) and kept on ice for 30 min. Cell lysates were sonicated at 4°C for 10 min (30 s ON, 30 s OFF) at medium frequency (Bioruptor Diagenode). Then, the lysates were centrifugated for 10 min at 4°C at maximum speed and the supernatants were kept as the samples. Extracts were resolved on pre-cast polyacrylamide gel cassettes (NuPAGE 4 to 12% Bis-Tris) (Invitrogen) and 1× NuPAGE MOPS SDS Running Buffer and transferred into nitrocellulose membrane (Amersham) in 20 mM phosphate transfer buffer (pH 6.7). The membrane was blocked in 5% skim milk in PBST buffer (1× PBS, 0.2% Tween 20) and incubated overnight at 4°C with the primary antibody (see Table 3). Membranes were washed twice for 5 min in PBST, incubated with appropriate secondary antibody IRDye (Li-Cor) in PBST, washed twice for 10 min in PBST, once for 10 min in PBS, and then imaged on Odyssey Imaging System. The used antibodies are listed in Table 3.

### Transcriptome analysis and bioinformatics

RNA was isolated as described above. Two or three independent biological replicates were sequenced depending on the cell conditions. Libraries were generated using the Ion AmpliSeq Transcriptome Human Gene Expression Kit (Ion Torrent, A24325). Sequencing was performed on an Ion S5 sequencer (Ion Torrent). The reads were analyzed using the plugin AmpliseqRNA of Torrent Suite (release 5.10) and mapped on the panel of human AmpliseqRNA which reveals the expression of 20813 genes. Out of these, 18,185 genes were uniquely mapped on human reference gene names (retrieved from ENSEMBL_102 release).

Using the raw count table generated, genes having low counts were filtered out on the basis of the log_2_ of counts per million (logCPM). We kept genes that have at least a 1 logCPM mean expression level in at least one of the experimental conditions, leaving 12,060 expressed genes for all the downstream analyses. The differential expression analysis of the filtered data was performed using the edgeR package of R and the limma-trend method described in (*40*). For each experimental setting, DEGs were further analyzed for all the available enrichments. Enrichment analysis and visualization of the DEGs were performed using the clusterProfiler R package (*41*), using Gene Ontologies, DOSE, the MSigDB database, and GSEA (*42*).

### Statistical analyses

Statistical analyses were carried out using Excel and R. Data are represented as means ± SEM as described in the figure legend. Graphs are prepared using Excel and R. Double tail *t* test was used for statistical analysis; *P < 0.05, **P < 0.01, and ***P < 0.001.
