# Supplementary material for: SETDB1 modulates the TGFβ response in Duchenne muscular dystrophy myotubes
Source: Sci Adv. 2024 May 1;10(18):eadj8042. doi: 10.1126/sciadv.adj8042 (PMC11062573; doi:10.1126/sciadv.adj8042)
Supplement: Supplementary file 1 — Figs. S1 to S7 Tables S1 to S4 [file sciadv.adj8042_sm.pdf]

Science Advances
AAAS

Supplementary Materials for

SETDB1 modulates the TGFβ response in Duchenne muscular dystrophy myotubes

Alice Granados *et al.*

Corresponding author: Slimane Ait-Si-Ali, slimane.aitsiali@u-paris.fr; Sonia Albini, salbini@genethon.fr

Sci. Adv. **10**, eadj8042 (2024)
DOI: 10.1126/sciadv.adj8042

This PDF file includes:

Figs. S1 to S7
Tables S1 to S4

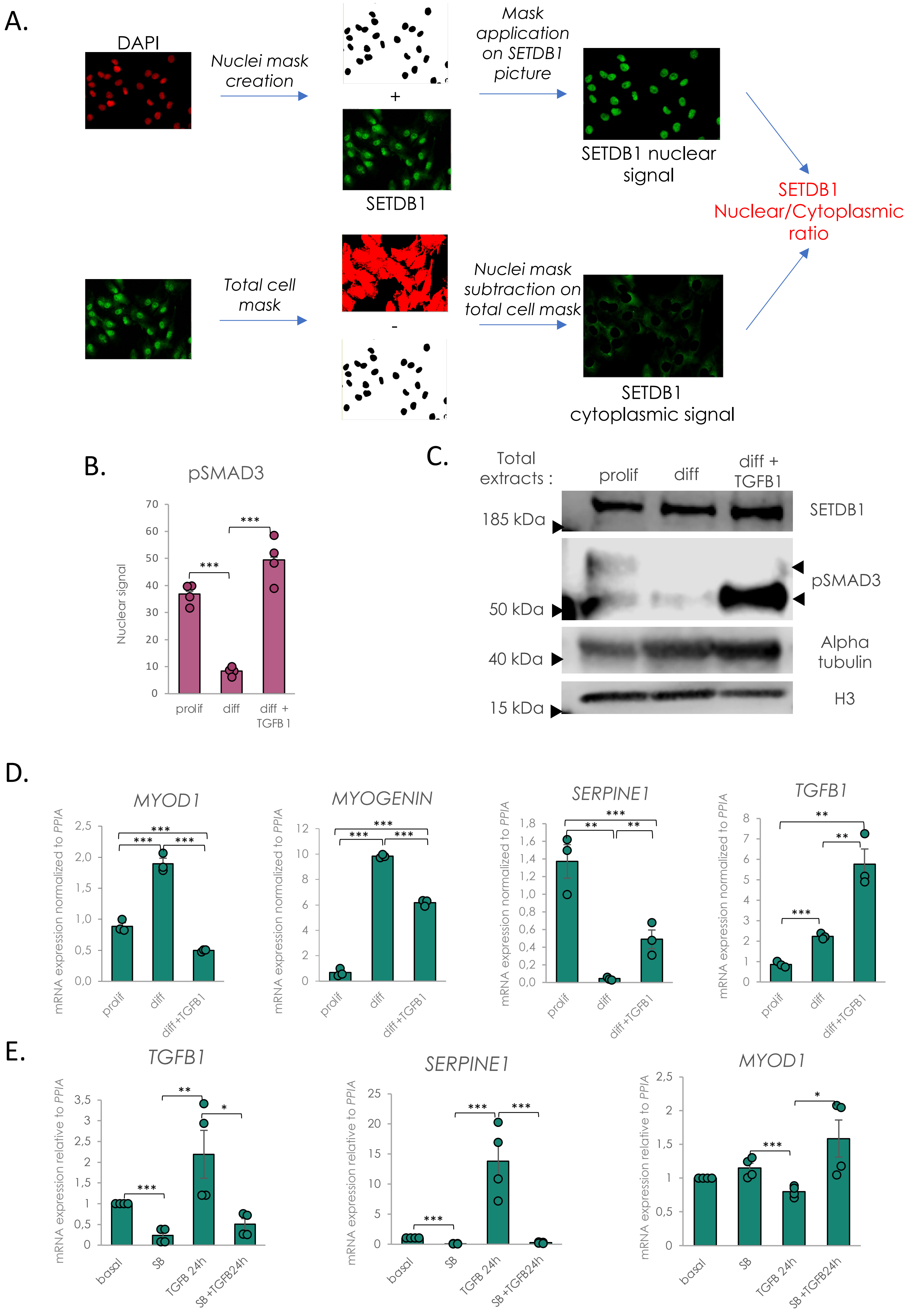
A.
DAPI
Nuclei mask creation
+
SETDB1
Mask application on SETDB1 picture
SETDB1 nuclear signal
SETDB1 Nuclear/Cytoplasmic ratio
Total cell mask
-
Nuclei mask subtraction on total cell mask
SETDB1 cytoplasmic signal
B.
pSMAD3
Nuclear signal
70
60
50
40
30
20
10
0
***
***
prolif
diff
diff + TGFB1
C.
Total extracts :
prolif
diff
diff + TGFB1
185 kDa
SETDB1
pSMAD3
50 kDa
40 kDa
Alpha tubulin
15 kDa
H3
D.
MYOD1
mRNA expression normalized to PPIA
2,5
2,0
1,5
1,0
0,5
0,0
***
***
***
prolif
diff
diff+TGFB1
MYOGENIN
mRNA expression normalized to PPIA
12
10
8
6
4
2
0
***
***
***
prolif
diff
diff+TGFB1
SERPINE1
mRNA expression normalized to PPIA
1,8
1,6
1,4
1,2
1,0
0,8
0,6
0,4
0,2
0,0
***
**
**
prolif
diff
diff+TGFB1
TGFB1
mRNA expression normalized to PPIA
8
7
6
5
4
3
2
1
0
**
**
***
prolif
diff
diff+TGFB1
E.
TGFB1
mRNA expression relative to PPIA
3,5
3
2,5
2
1,5
1
0,5
0
**
*
***
basal
SB
TGFB 24h
SB+TGFB24h
SERPINE1
mRNA expression relative to PPIA
25
20
15
10
5
0
***
***
***
basal
SB
TGFB 24h
SB+TGFB24h
MYOD1
mRNA expression relative to PPIA
2,5
2
1,5
1
0,5
0
*
***
basal
SB
TGFB 24h
SB+TGFB24h

Figure S1: SETDB1 localization depends on TGFβ/SMAD pathway activation during muscle terminal differentiation

A. Scheme of microscopy analysis and nuclear/cytoplasmic signal quantification. Nuclear masks were setup from DAPI pictures and applied on SETDB1 pictures and total cell masks were selected on SETDB1 pictures. Cytoplasmic quantification was performed by subtracting nuclei mask from total cell masks. **B.** Quantification of phospho-SMAD3 nuclear fluorescence in proliferating myoblasts, myotubes treated or not with TGFβ1. **C.** Protein levels of SETDB1 and phospho-SMAD3 in total extracts of myoblasts and myotubes treated or not with TGFβ1.

D. RT-qPCR of early myogenic (*MYOD1, Myogenin*) and TGFβ-related genes (*TGFB1, SERPINE1*) in myoblasts and myotubes treated or not with TGFβ1. **E.** RT-qPCR of early myogenic marker *MYOD1* and TGFβ-related genes (*SERPINE1, TGFB1*) in myotubes treated or not with TGFβ1 and/or its inhibitor SB-431542.

For all panels: Statistics were performed on ≥3 biological replicates (>100 nuclei for immunostaining quantification) and data are represented as average +/- SEM *p<0.05; **p<0.01; ***p<0.001 (unpaired Student's t test).

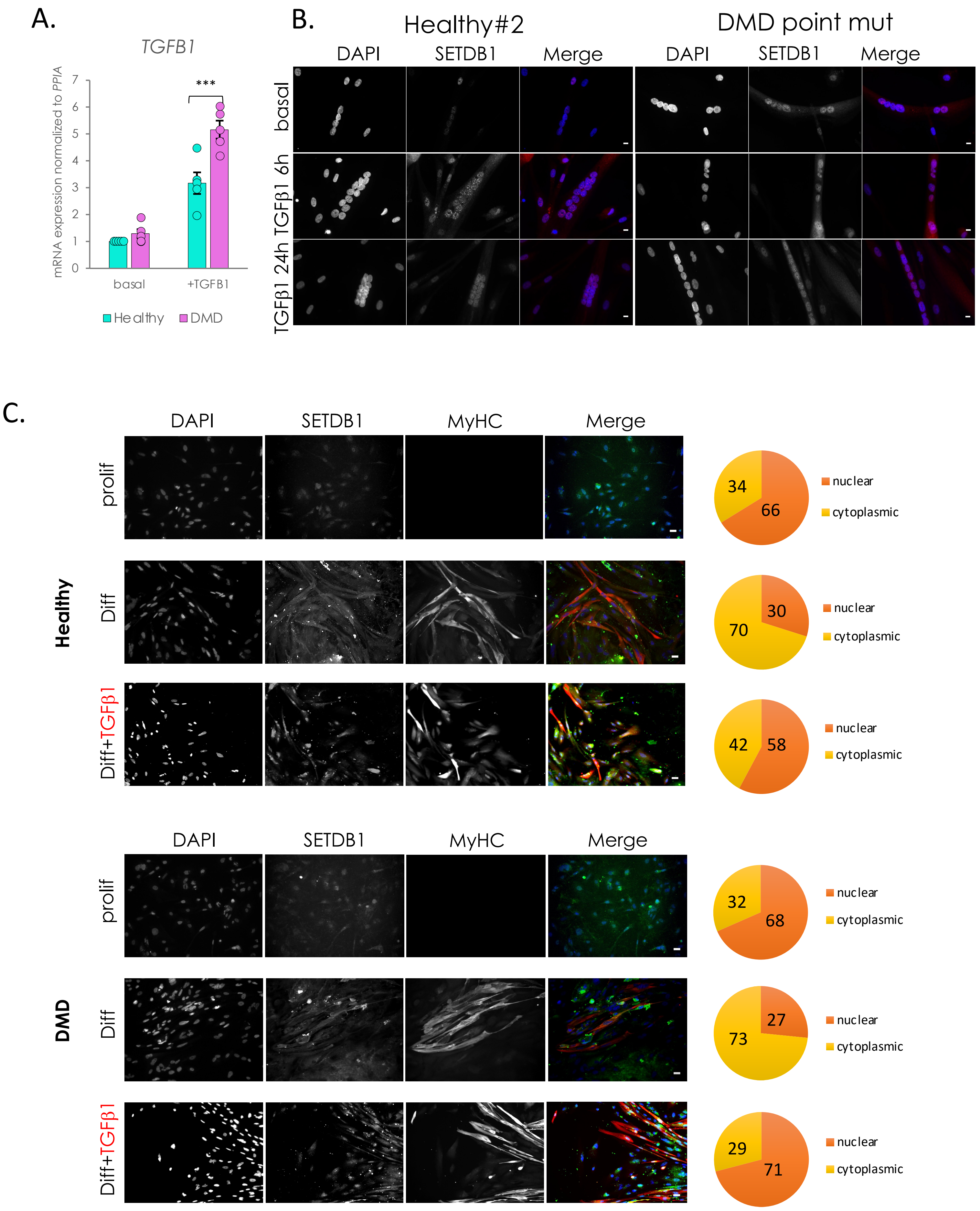

A.
TGFB1
mRNA expression normalized to PPIA
7
6
5
4
3
2
1
0
***
basal
+TGFB1
Healthy
DMD
B.
Healthy#2
DMD point mut
DAPI
SETDB1
Merge
DAPI
SETDB1
Merge
basal
TGFβ1 6h
TGFβ1 24h
C.
DAPI
SETDB1
MyHC
Merge
Healthy
prolif
Diff
Diff+TGFβ1
34
66
nuclear
cytoplasmic
30
70
nuclear
cytoplasmic
42
58
nuclear
cytoplasmic
DAPI
SETDB1
MyHC
Merge
DMD
prolif
Diff
Diff+TGFβ1
32
68
nuclear
cytoplasmic
27
73
nuclear
cytoplasmic
29
71
nuclear
cytoplasmic

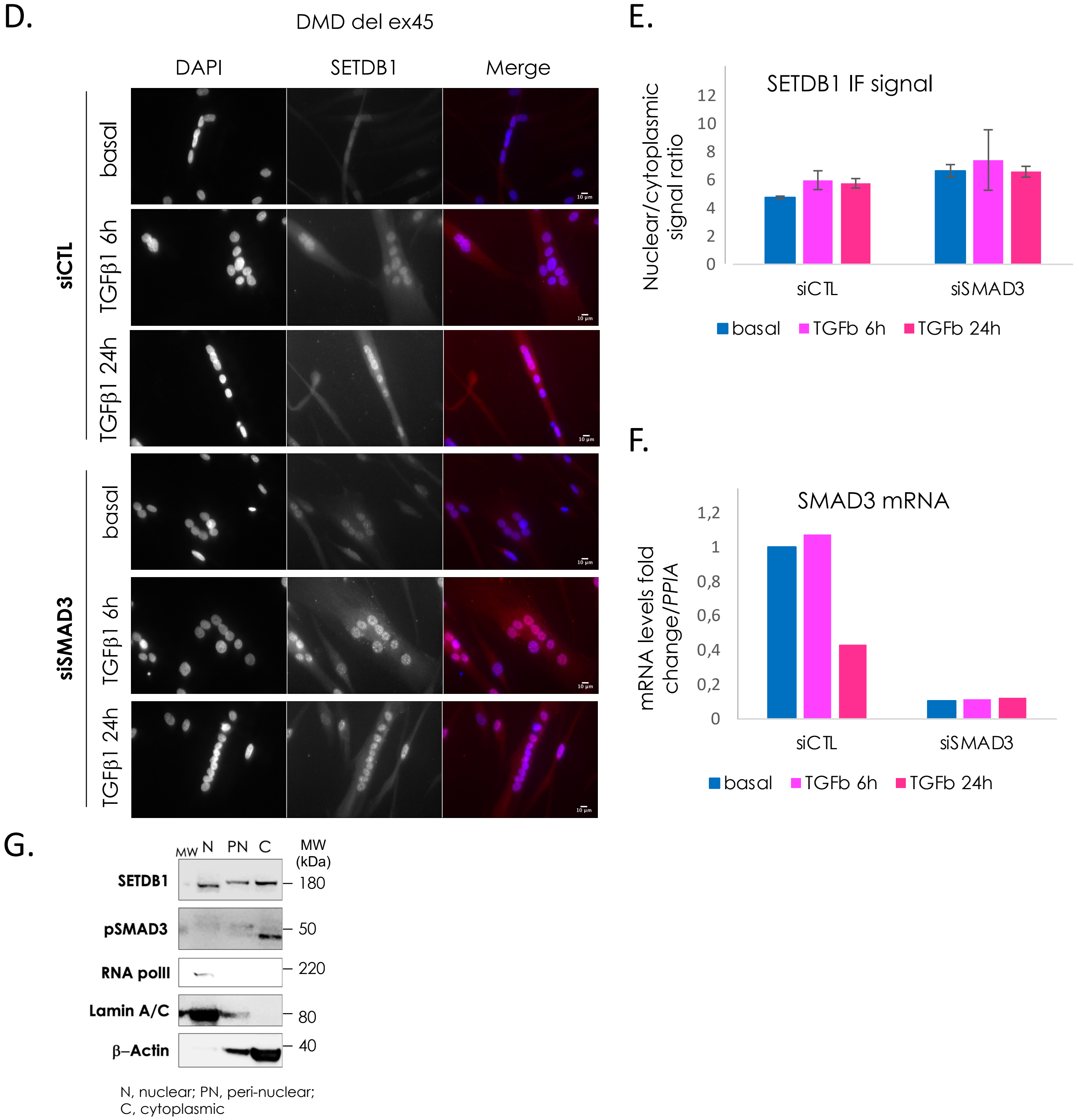

Figure S2: SETDB1 translocate into muscle cell nuclei in response to TGFβ/SMAD pathway activation and show more persistent nuclear signal in DMD myotubes irrespectively of the type of DMD mutation

A. RT-qPCR of *TGFB1* shows a higher response of DMD myotubes to TGFβ1 treatment as compared to WT cells. **B.** Immunostaining of SETDB1 (red) in healthy#2 and DMD point mutation myotubes. Nuclei were stained with DAPI (blue). Scale bar, 10 μM. **C.** SETDB1 and MyHC immunostaining in proliferating muscle cells and in myotubes treated or not with TGFβ1 derived from iPSCs of healthy or DMD individuals. Nuclei were stained with DAPI (blue). Scale bar, 10 μM. Diagrams represent the percentage of SETDB1 signal intensity measure in nuclei and cytoplasm of the cells. **D-F.** DMD del ex45 differentiating myotubes (48 h differentiation) were transfected with control scrambled (siCTL) or SMAD3 siRNA (siSMAD3). 2 days later, myotubes were treated with TGFβ and after 1 day, myotubes were subjected to different assays, as follows: **D.** Immunostaining of SETDB1 (red), and nuclei staining with DAPI (blue). Scale bar, 10 μm. **E.** Quantification of SETDB1 (n=2) nuclear/cytoplasmic IF signal ratio. **F.** RT-qPCR of *SMAD3* mRNA in control siRNA (siCTL) and siRNA against SMAD3 (siSMAD3) conditions to check the siRNA efficiency (representative of n=2). **G.** Western blot showing SETDB1 and pSMAD3 protein migration profiles in nuclear (N), peri-nuclear (PN) and cytoplasmic (C) fractions of healthy myotubes. RNA polymerase II and Lamin A/C were used as controls for nuclear fraction and beta-Actin as a control of cytoplasmic fraction.

For all panels: Statistics were performed on ≥3 biological replicates (>100 nuclei for immunostaining quantification) and data are represented as average +/- SEM *p<0.05; **p<0.01; ***p<0.001 (unpaired Student's t test).

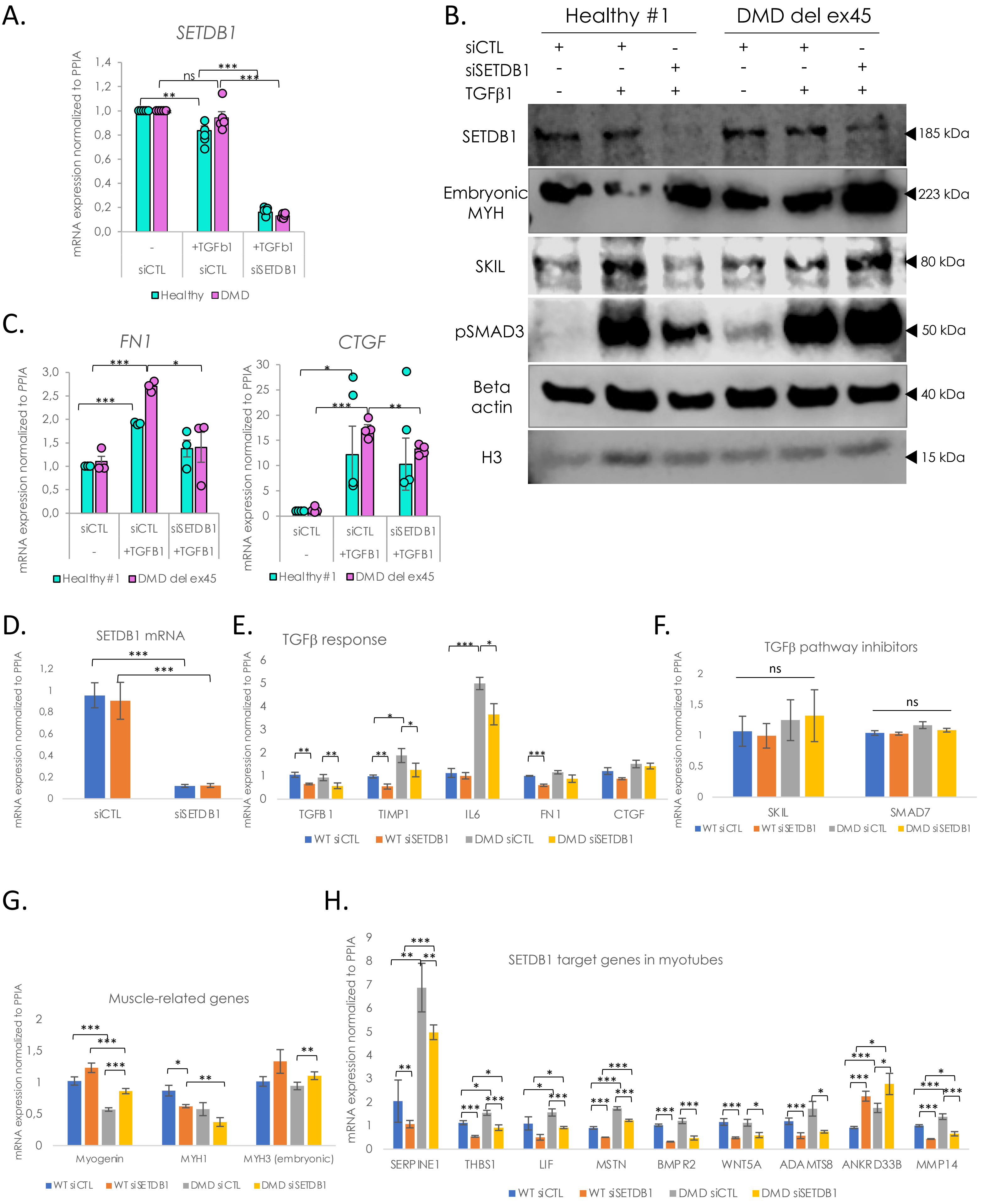

A.
SETDB1
mRNA expression normalized to PPIA
1,4
1,2
1,0
0,8
0,6
0,4
0,2
0,0
ns
***
***
**
-
+TGFb1
+TGFb1
siCTL
siCTL
siSETDB1
Healthy
DMD
B.
Healthy #1
DMD del ex45
siCTL
siSETDB1
TGFβ1
SETDB1
Embryonic MYH
SKIL
pSMAD3
Beta actin
H3
185 kDa
223 kDa
80 kDa
50 kDa
40 kDa
15 kDa
C.
FN1
CTGF
mRNA expression normalized to PPIA
siCTL
siCTL
siSETDB1
-
+TGFB1
+TGFB1
Healthy#1
DMD del ex45
D.
SETDB1 mRNA
siCTL
siSETDB1
E.
TGFβ response
TGFB1
TIMP1
IL6
FN1
CTGF
WT siCTL
WT siSETDB1
DMD siCTL
DMD siSETDB1
F.
TGFβ pathway inhibitors
SKIL
SMAD7
G.
Muscle-related genes
Myogenin
MYH1
MYH3 (embryonic)
H.
SETDB1 target genes in myotubes
SERPINE1
THBS1
LIF
MSTN
BMPR2
WNT5A
ADAMTS8
ANKRD33B
MMP14

Figure S3: Efficient siRNA-mediated SETDB1 knockdown in TGFβ-treated DMD myotubes leads to a decrease in TGFβ target gene expression and an increase in pro-myogenic factors *SKIL* and *MYH3*

A. RT-qPCR of *SETDB1* shows an acute decrease (>80%) of *SETDB1* expression upon siRNAs-mediated silencing. *SETDB1* expression decreases upon TGFβ1 treatment in healthy myotubes but not in DMD myotubes. **B.** Western blot showing protein levels of SETDB1, SKIL, embryonic MYH and phospho-SMAD3 in healthy *versus* DMD myotubes upon SETDB1 silencing and TGFβ1 treatment. **C.** RT-qPCR of TGFβ/SMADs pathway known targets *FN1* and *CTGF* in healthy and DMD myotubes +/- siSETDB1 +/- TGFβ1. TGFβ-related gene expression is decreased in SETDB1-silenced *versus* siCTL DMD myotubes in response to TGFβ1. **D-H.** RT-qPCR of *SETDB1* (**D**), TGFβ known target genes *TGFB1*, *TIMP1*, *IL6*, *FN1* and *CTGF* (**E**), of TGFβ pathway inhibitors *SKIL* and *SMAD7* (**F**), muscle differentiation genes *Myogenin*, *MYH1* and *MYH3* (**G**) and of SETDB1 target genes such as *SERPINE1*, *THBS1*, *LIF*, *MSTN*, *BMPR2*, *WNT5A*, *ADAMTS8*, *ANKRD33B* and *MMP14* (**H**) in healthy and DMD myotubes +/- siSETDB1 in basal condition (without TGFβ treatment).

For all panels: Statistics were performed on ≥3 biological replicates and data are represented as average +/- SEM *p<0.05; **p<0.01; ***p<0.001 (unpaired Student's t test).

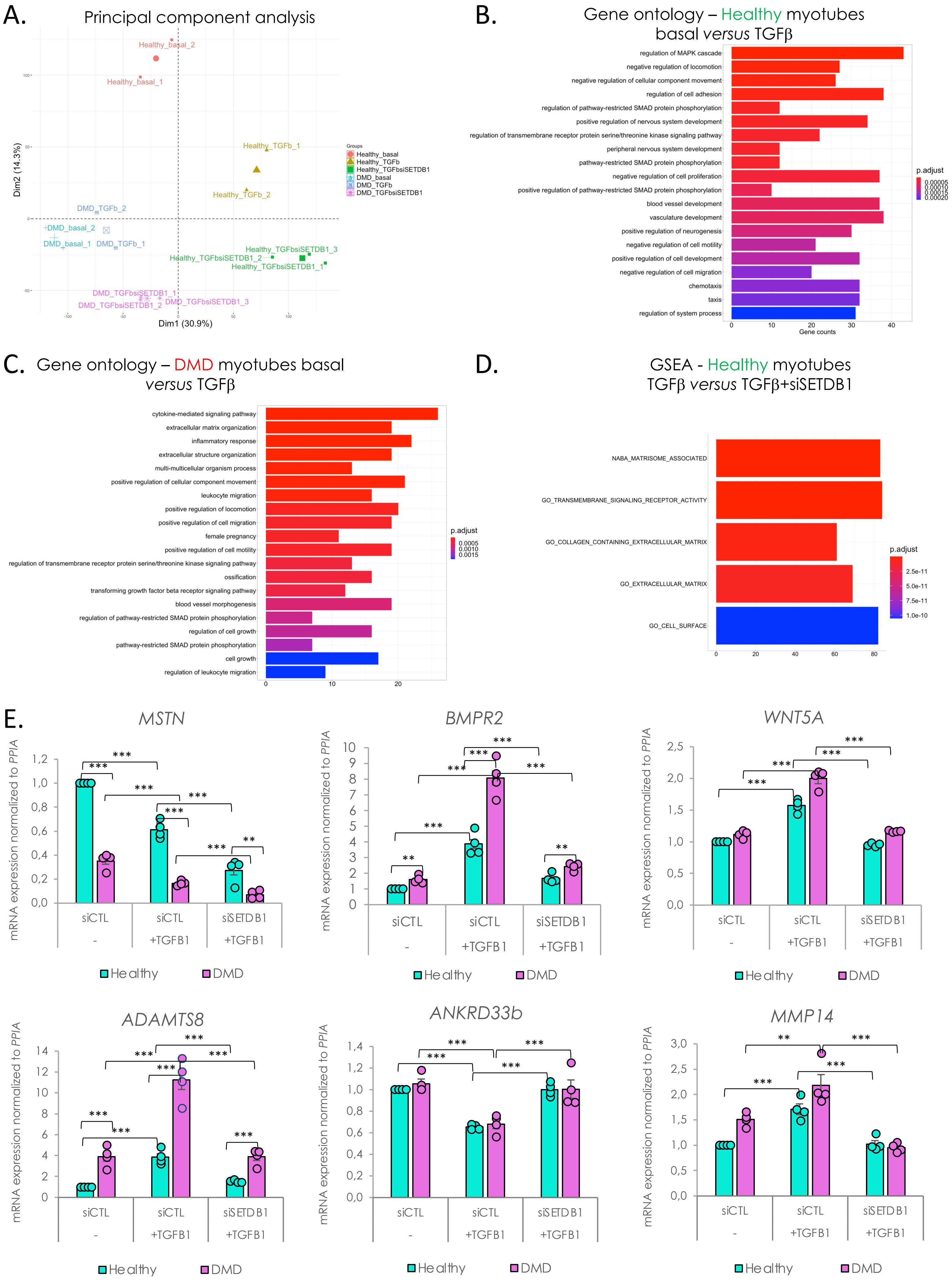

A.
Principal component analysis
Healthy_basal_2
Healthy_basal_1
Healthy_TGFb_1
Healthy_TGFb_2
DMD_TGFb_2
DMD_basal_2
DMD_basal_1
DMD_TGFb_1
Healthy_TGFbsiSETDB1_3
Healthy_TGFbsiSETDB1_2
Healthy_TGFbsiSETDB1_1
DMD_TGFbsiSETDB1_1
DMD_TGFbsiSETDB1_2
DMD_TGFbsiSETDB1_3
Dim1 (30.9%)
Dim2 (14.3%)
Groups
Healthy_basal
Healthy_TGFb
Healthy_TGFbsiSETDB1
DMD_basal
DMD_TGFb
DMD_TGFbsiSETDB1
B.
Gene ontology – Healthy myotubes basal versus TGFβ
regulation of MAPK cascade
negative regulation of locomotion
negative regulation of cellular component movement
regulation of cell adhesion
regulation of pathway-restricted SMAD protein phosphorylation
positive regulation of nervous system development
regulation of transmembrane receptor protein serine/threonine kinase signaling pathway
peripheral nervous system development
pathway-restricted SMAD protein phosphorylation
negative regulation of cell proliferation
positive regulation of pathway-restricted SMAD protein phosphorylation
blood vessel development
vasculature development
positive regulation of neurogenesis
negative regulation of cell motility
positive regulation of cell development
negative regulation of cell migration
chemotaxis
taxis
regulation of system process
Gene counts
p.adjust
0.00005
0.00010
0.00015
0.00020
C.
Gene ontology – DMD myotubes basal versus TGFβ
cytokine-mediated signaling pathway
extracellular matrix organization
inflammatory response
extracellular structure organization
multi-multicellular organism process
positive regulation of cellular component movement
leukocyte migration
positive regulation of locomotion
positive regulation of cell migration
female pregnancy
positive regulation of cell motility
regulation of transmembrane receptor protein serine/threonine kinase signaling pathway
ossification
transforming growth factor beta receptor signaling pathway
blood vessel morphogenesis
regulation of pathway-restricted SMAD protein phosphorylation
regulation of cell growth
pathway-restricted SMAD protein phosphorylation
cell growth
regulation of leukocyte migration
p.adjust
0.0005
0.0010
0.0015
D.
GSEA - Healthy myotubes TGFβ versus TGFβ+siSETDB1
NABA_MATRISOME_ASSOCIATED
GO_TRANSMEMBRANE_SIGNALING_RECEPTOR_ACTIVITY
GO_COLLAGEN_CONTAINING_EXTRACELLULAR_MATRIX
GO_EXTRACELLULAR_MATRIX
GO_CELL_SURFACE
p.adjust
2.5e-11
5.0e-11
7.5e-11
1.0e-10
E.
MSTN
BMPR2
WNT5A
ADAMTS8
ANKRD33b
MMP14
mRNA expression normalized to PPIA
siCTL
-
siCTL
+TGFB1
siSETDB1
+TGFB1
Healthy
DMD

F.

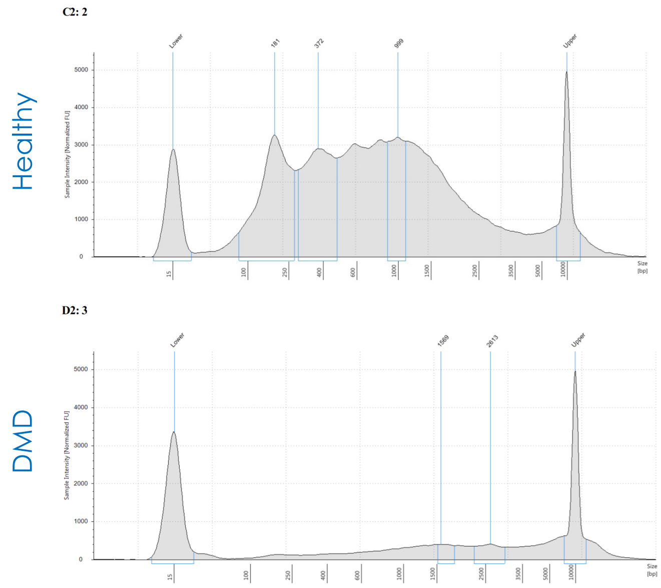

Figure S4: Healthy and DMD myotubes respond differently to TGFβ/SMAD pathway activation but display some SETDB1 target gene signatures in common

A. Principal component analysis of the filtered (see Materials & Methods) RNA-seq data, depicting a well grouping of all the samples for each of the 6 experimental conditions of the study. The variability captured by the first PC corresponds to the healthy vs. DMD conditions and the one of the second PC to the TGFβ treatment. **B.** Gene Ontology enrichment of DEGs from healthy myotubes basal *versus* TGFβ comparison. **C.** Gene Ontology enrichment of DEGs from DMD myotubes basal *versus* TGFβ comparison. **D.** Gene Set Enrichment Analysis (GSEA) of DEGs from healthy myotubes TGFβ +/- siSETDB1. **E.** Validation by RT-qPCR of genes coding for proteins involved in ECM-remodeling (*ADAMTS8, MMP14*), TGFβ/BMP and Wnt pathway (*MSTN, BMPR2, WNT5A*) and unknown function but predicted as regulator of muscle differentiation (*ANKRD33B*). **F.** TapeStation profile of ATAC libraries from healthy or DMD myotubes prepared using the ATAC-seq kit from Diagenode (ref: C01080001).

For all panels: Statistics were performed on ≥3 biological replicates and data are represented as average +/- SEM *p<0.05; **p<0.01; ***p<0.001 (unpaired Student's t test).

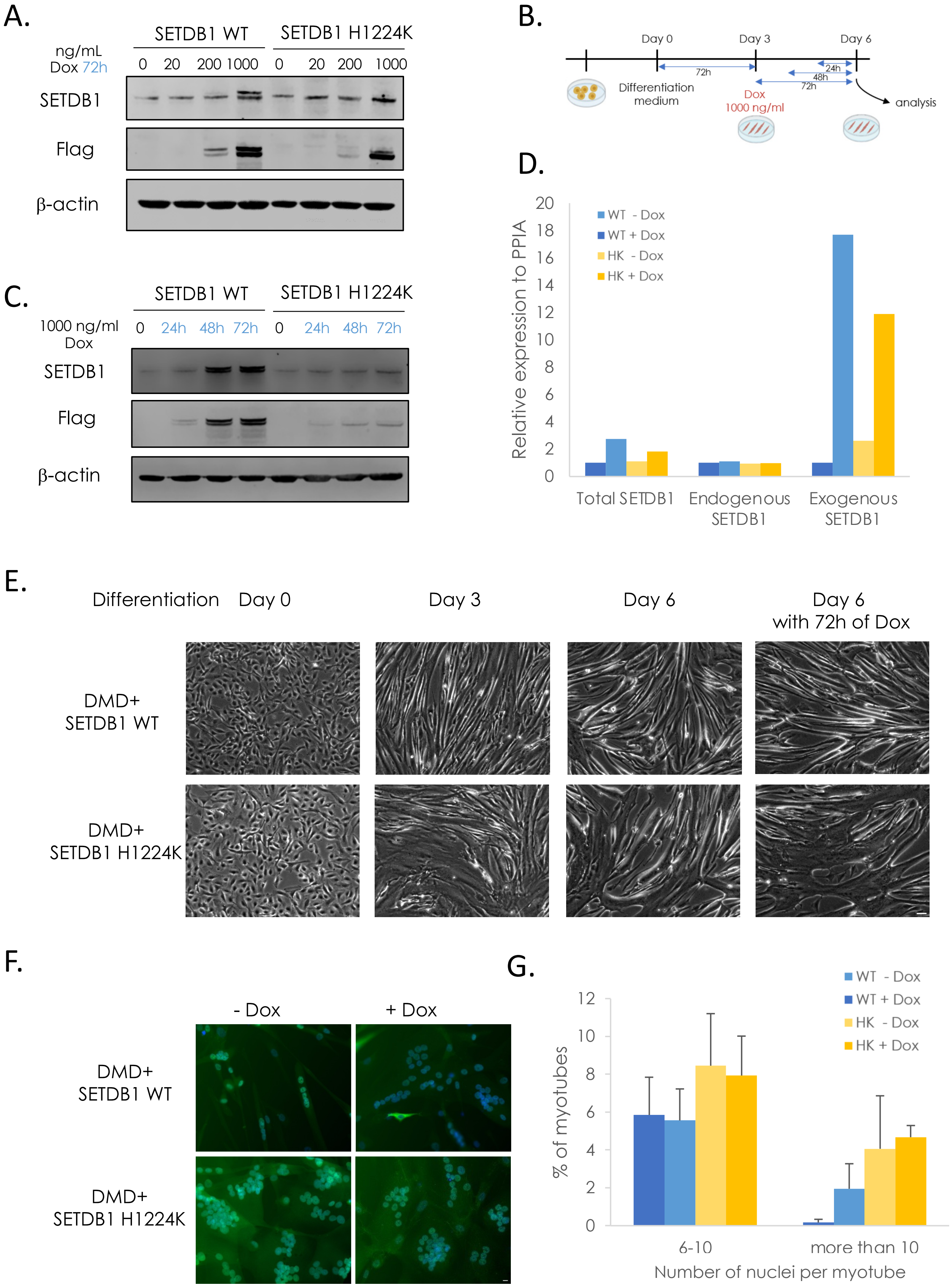
A.
SETDB1 WT
SETDB1 H1224K
ng/mL
Dox 72h
0 20 200 1000 0 20 200 1000
SETDB1
Flag
β-actin
B.
Day 0
Day 3
Day 6
72h
24h
48h
72h
Differentiation
medium
Dox
1000 ng/ml
analysis
C.
SETDB1 WT
SETDB1 H1224K
1000 ng/ml
Dox
0 24h 48h 72h 0 24h 48h 72h
SETDB1
Flag
β-actin
D.
Relative expression to PPIA
20
18
16
14
12
10
8
6
4
2
0
WT - Dox
WT + Dox
HK - Dox
HK + Dox
Total SETDB1
Endogenous
SETDB1
Exogenous
SETDB1
E.
Differentiation
Day 0
Day 3
Day 6
Day 6
with 72h of Dox
DMD+
SETDB1 WT
DMD+
SETDB1 H1224K
F.
- Dox
+ Dox
DMD+
SETDB1 WT
DMD+
SETDB1 H1224K
G.
WT - Dox
WT + Dox
HK - Dox
HK + Dox
% of myotubes
12
10
8
6
4
2
0
6-10
more than 10
Number of nuclei per myotube

Figure S5: Catalytic-dead SETDB1 induced a strong differentiation phenotype even at low expression levels.

A. Western blot (WB) on DMD myotube protein extracts after 72 hours of treatment with the indicated different concentrations of doxycycline, showing protein levels of SETDB1 (endogenous and exogenous), FLAG (exogenous SETDB1), and β-actin used as loading controls. **B.** Diagram of the experimental plan. **C.** WB on DMD myotube protein extracts at different time points post-treatment with 1000 ng/mL doxycycline. **D.** Validation by RT-qPCR of SETDB1 total, endogenous and exogenous mRNA after 72h of doxycycline treatment. **E.** Polyclonal DMD myoblasts differentiation observed by phase contrast light microscopy. Scale bar, 50 μM. **F.** Immunostaining of SETDB1 (green). Nuclei were stained with DAPI (blue). Scale bar, 10 μm. **G.** Quantification of nuclei/myotubes in the presence or absence of WT SEDTB1 and catalytic-dead mutant H1224K SETDB1.

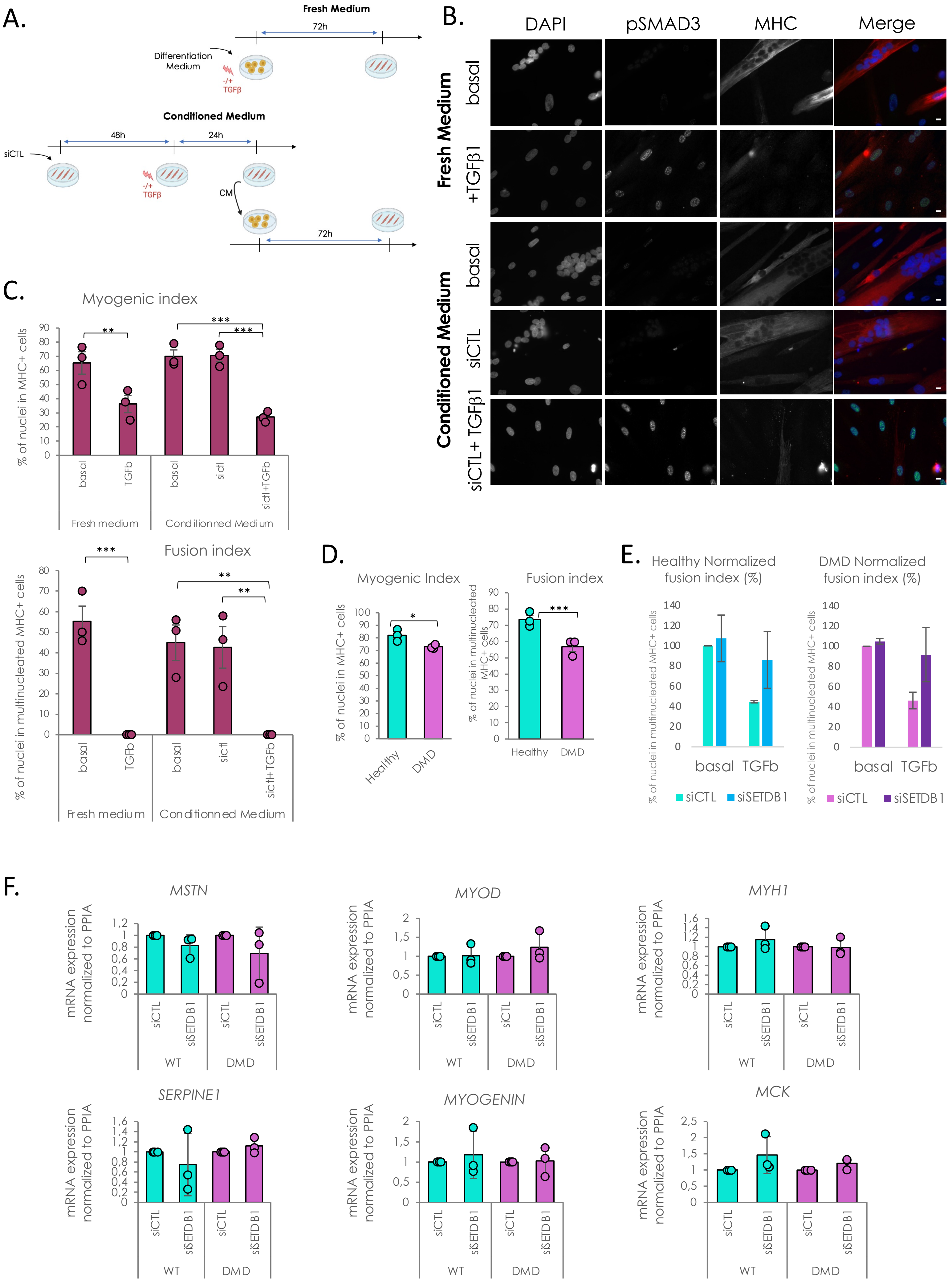
A.
Fresh Medium
72h
Differentiation Medium
-/+ TGFβ
Conditioned Medium
48h
24h
siCTL
-/+ TGFβ
CM
72h
B.
DAPI
pSMAD3
MHC
Merge
Fresh Medium
basal
+TGFβ1
Conditioned Medium
basal
siCTL
siCTL+ TGFβ1
C.
Myogenic index
% of nuclei in MHC+ cells
basal
TGFb
basal
sictl
sictl+TGFb
Fresh medium
Conditionned Medium
**
***
***
Fusion index
% of nuclei in multinucleated MHC+ cells
basal
TGFb
basal
sictl
sictl+ TGFb
Fresh medium
Conditionned Medium
***
**
**
D.
Myogenic Index
% of nuclei in MHC+ cells
Healthy
DMD
*
Fusion index
% of nuclei in multinucleated MHC+ cells
Healthy
DMD
***
E.
Healthy Normalized fusion index (%)
DMD Normalized fusion index (%)
% of nuclei in multinucleated MHC+ cells
basal
TGFb
siCTL
siSETDB1
F.
MSTN
MYOD
MYH1
SERPINE1
MYOGENIN
MCK
mRNA expression normalized to PPIA
siCTL
siSETDB1
WT
DMD

Figure S6: TGFβ/SMAD pathway activation leads to fusion defects in muscle cells

A. Diagram of the experimental design. **B.** Immunofluorescence of pSMAD3 (green) and MHC (red) in myoblasts differentiated in fresh or conditioned medium +/- TGFβ1. Nuclei were stained with DAPI (blue). Scale bar, 10 μM. **C.** Myogenic and fusion index of the myoblasts differentiated in fresh or conditioned medium +/- TGFβ1. **D.** Raw myogenic and fusion index in basal conditioned medium show difference in differentiation rate between healthy and DMD myotubes.

E. Quantification of fusion index after 6 days of differentiation in conditioned medium produced by healthy or DMD myotubes +/- siSETDB1 +/- TGFβ1. **F.** RT-qPCR of pro-fibrotic genes, *MSTN* and *SERPINE1*, and muscle-related genes *MYOD1*, *Myogenin*, *MYH1* and *MCK* after 3 days of differentiation in conditioned medium produced by healthy or DMD myotubes +/- siSETDB1.

For all panels: Statistics were performed on ≥3 biological replicates (>100 nuclei for immunostaining quantification) and data are represented as average +/- SEM $^*p<0.05$; $^{**}p<0.01$; $^{***}p<0.001$ (unpaired Student's t test).

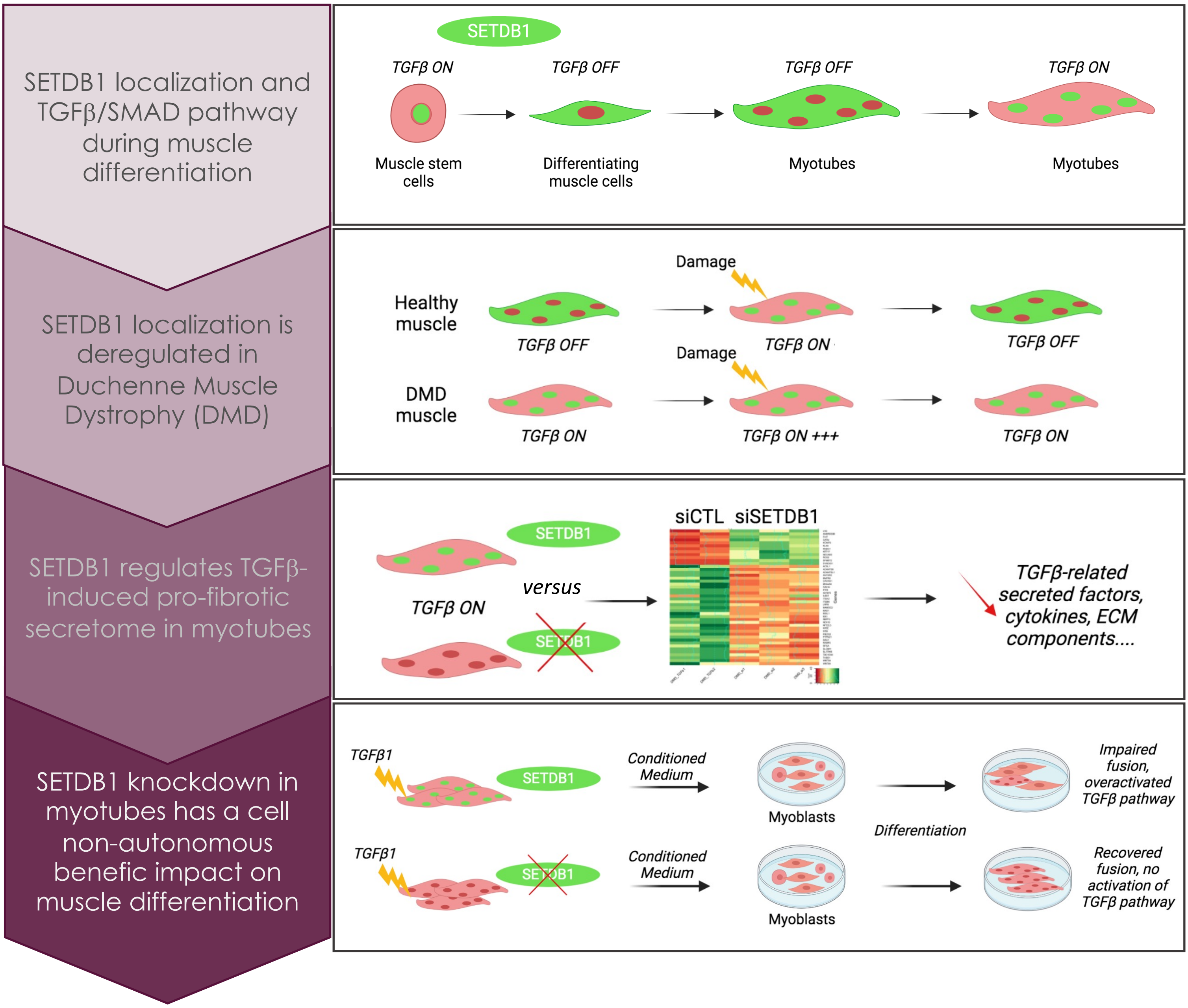

Figure S7: Graphical Abstract

- TGFβ induces nuclear accumulation of SETDB1 in healthy myotubes
- SETDB1 is enriched in DMD myotube nuclei with intrinsic TGFβ pathway overactivation
- SETDB1 LOF in DMD myotubes attenuates TGFβ-induced pro-fibrotic response
- Secretome of TGFβ-treated DMD myotubes with SETDB1 LOF is less deleterious on myoblast differentiation

Table S1: Top DEGs, + *versus* – TGFβ1 in healthy myotubes

Genes	LogFC	Adj p-value	Genes	LogFC	Adj p-value	Genes	LogFC	Adj p-value
TAGLN2	2,117414122	0,0000029	FZD1	-1,709895607	0,000820576	CYGB	1,91232727	0,004636356
PMAIP1	1,822825158	0,0000295	CPT1A	1,892601801	0,000847677	CHRNA9	2,131251441	0,004655553
SPSB1	1,954444533	0,0000431	FLRT3	-1,666029718	0,000907678	HES6	1,511256537	0,004686457
LEFTY2	2,378015571	0,0000633	SPOCD1	2,072190972	0,000955143	BHLHE40	1,577516443	0,004688974
MYL10	2,775733677	0,000102766	KY	1,741592873	0,000987349	DUSP15	1,504729237	0,004688974
NGF	1,657626522	0,000102766	MMP24	1,785794877	0,001060517	PRTG	-1,939105695	0,004688974
HSD11B2	2,056381007	0,00014734	PTPRN	1,572910987	0,001060517	TAS2R50	-1,617874683	0,004707551
CPEB1	2,008260893	0,00014734	SPHK1	1,553357986	0,001060517	CAPG	1,617433777	0,004761555
GRIK3	1,897772958	0,00014734	SLC6A15	-1,604281321	0,001060517	IL15	1,632420492	0,004800381
CAMK2B	1,66141848	0,00014734	RGS10	1,775216817	0,001091293	RCSD1	1,50536248	0,005066626
LIF	1,609459933	0,00014734	ACAP1	-1,518147984	0,001091293	BCHE	-1,514123565	0,00508091
FAS	1,65594723	0,000176389	CDH22	1,952879439	0,001160065	HAPLN1	-1,939131798	0,005275934
COMP	3,079879046	0,000193172	VDR	2,38037933	0,001185628	TRAPPC11	-1,683727235	0,005288437
KRT17	2,249228221	0,000193172	MADCAM1	2,012788312	0,001218815	E2F2	-1,769263425	0,005304981
CYSLTR1	-1,686154285	0,000193172	IL11	1,720981756	0,00125013	ABHD1	-1,604574292	0,005424992
EFNA5	-1,539212907	0,000193172	ATF3	1,505914322	0,001406572	EDN3	-2,42948784	0,005452562
CBFA2T3	1,585761128	0,00021232	BNC1	1,727769999	0,001463008	KCNG3	-2,006427044	0,005469823
TINAGL1	2,040560934	0,000214261	UGT8	-2,091670121	0,001463008	MT2A	2,078060952	0,005630917
ST6GALNAC2	2,621131534	0,000278225	MDGA1	-1,57495199	0,001500196	MMRN1	-5,596523239	0,005630917
MYL7	1,928998074	0,000278225	LRRN3	-2,023789769	0,001564602	NPPB	2,22275891	0,005782479
RAB11FIP4	1,68799875	0,000278225	APCDD1L	1,573817321	0,001603986	SFRP1	-1,766514568	0,005851596
CADM2	-1,756947008	0,000278225	LEFTY1	2,948081363	0,001646694	NUPR1	2,251896144	0,006190195
KCNH3	1,526606981	0,000294724	INSC	1,984068026	0,001654329	MAPK10	-1,765559007	0,006190195
SH2D4A	1,542140036	0,000304292	FGFBP3	-1,960631592	0,001750743	TENM1	-1,823947329	0,006245981
BMP8A	1,66627264	0,000311971	LRP2	-2,613936968	0,001789815	ABCA7	-1,6412153	0,006245981
UBASH3B	1,556460437	0,000311971	KCNK3	2,416533797	0,001890051	ADAMTS3	-1,554488887	0,006363145
GAL	2,095209135	0,000339293	DSCAML1	2,603034024	0,001909552	SPATA22	1,86416685	0,006490852
FZD5	-2,217424853	0,000339293	LPAR4	-2,840036833	0,002027283	MAB21L2	-2,344727736	0,006490852
ERBB4	-1,608195924	0,000339293	BMP6	1,797677275	0,002067634	PREX2	-1,895214593	0,00654097
JUNB	1,651100338	0,000363745	DRD2	1,911310104	0,002119699	LRRIQ1	-2,230304268	0,006747866
SYTL1	2,38078558	0,000381256	INHBA	1,943531045	0,002198855	PLXDC1	1,720148106	0,006814196
APOBEC3C	1,532547029	0,000381256	HHIPL2	-3,371136543	0,002252125	CBR1	2,746093702	0,006991214
SNAI2	1,80924925	0,000391447	CRLF1	1,941126358	0,002294054	PTPRZ1	-2,289835098	0,006997138
RASL10A	1,877442599	0,000415202	ADAMTSL2	1,594546261	0,002388538	GATA3	1,58890351	0,007566377
PDK4	2,83974063	0,00043132	FZD3	-1,5642083	0,00246262	SLC6A3	-1,725541298	0,007724626
FBLN1	-1,588350862	0,000444297	MKI67	-1,620940441	0,002506658	EPHA4	-1,949119966	0,007737852
BMP3	-1,918899291	0,000449801	CXCR2	1,854163088	0,00256305	LTBP4	-1,510220625	0,008205986
LAMP5	1,686374304	0,000451403	FOSL1	1,871421395	0,002601514	RASD1	1,776910188	0,008344487
NDRG1	1,551820455	0,000451403	ZNF385B	-1,81912287	0,002601514	FAT4	-1,540323008	0,008352329
KCNT2	-1,6565026	0,00046383	SLC15A3	-1,776559618	0,002623801	TAS2R3	-2,803397109	0,008662833
PCDH18	-1,908015374	0,00048586	GPR26	2,554885167	0,002627384	FAM228B	1,920625724	0,008786462
C1QL3	2,750006723	0,000496238	PHLDA2	1,912747543	0,002627384	CGB5	1,997764466	0,008933737
SUMO4	-1,986991477	0,000506112	RRAD	2,053971838	0,002728733	DUSP2	1,65487497	0,008983737
ARHGDIB	1,850929182	0,000509686	FREM2	-1,565177808	0,002728733	ROBO1	-1,537260889	0,008983737
PCSK6	1,711984469	0,000509686	CAV1	1,914603656	0,002899024	GPR183	2,015068932	0,009086429
IRF1	1,590773748	0,000509686	WNK4	1,997056036	0,002931427	TTF1	-1,94369392	0,009313694
KIAA1211L	2,76892938	0,000517694	UGT3A1	-1,686489198	0,003345925	TAS2R31	-2,897454085	0,009353729
BBC3	1,535319531	0,000517694	MAPRE2	1,610130633	0,003380818	CD34	-3,228526136	0,009577181
ROBO2	-2,353637478	0,000534263	KDR	-1,538782171	0,003406179	ZNF493	-1,597943058	0,009582422
MSTN	-2,419217607	0,000572993	TFAP2C	1,719741735	0,003623613	OSGIN1	2,246915673	0,009631694
SFRP2	-2,547991562	0,00060178	PODN	1,685594321	0,003623613	ANK1	1,501084248	0,009652588
ZNF488	1,613702593	0,000668475	ASPH	-1,680643217	0,003696591	SCN3A	-1,978797967	0,009826333
CYP26C1	-2,60465348	0,000697753	NPR1	-1,93870055	0,00402619	SAC3D1	1,586634217	0,009949286
CDH6	-1,630541842	0,000707553	HES5	-5,038583814	0,004080276	TMEM168	-2,739006335	0,009993603
ADAMTS5	-1,875618352	0,000718764	SRGAP2	-1,614350913	0,004106754			
MX1	3,4674981	0,000725331	ISG15	1,862365951	0,004133911			
HMOX1	1,659405071	0,000725331	BIK	1,73069843	0,00451334			
HIST1H4D	-1,571617314	0,000814482	CACNG7	-1,885120997	0,004522138			
CDK5R2	1,65690867	0,000820304	IRS4	-2,193265101	0,004527926			
BMP5	-1,950914501	0,000820304	SLC7A8	-1,852506781	0,004527926			

Table S2: Top DEGs, + *versus* – TGFβ1 in DMD myotubes

Genes	LogFC	Adj p-value	Genes	LogFC	Adj p-value
TGFBI	2,75040849	5,16E-08	BNC1	2,22770933	0,001244942
IL32	2,163620694	2,67E-07	QPCT	1,556242386	0,001244942
LIF	2,335338653	4,04E-06	MYL10	1,972655907	0,00146367
TAGLN2	1,614876963	5,10E-06	C6orf15	1,678899961	0,001517905
LOX	2,910516076	1,49E-05	ACTN3	-1,617310124	0,00159821
IL11	3,500409887	1,56E-05	S100A11	1,542630473	0,001754586
ANGPTL4	2,505569257	1,56E-05	HMOX1	1,644698523	0,002038795
MARCH4	2,157892488	1,56E-05	PARP14	1,696207068	0,002076847
ITGA2	1,980632839	1,56E-05	FSTL3	2,029350269	0,002148251
APCDD1L	2,725494981	1,81E-05	NLRC5	3,843801654	0,00225234
CCL4	5,438924629	2,45E-05	WNK4	2,802240205	0,00225234
PRRT2	-1,567243856	3,16E-05	SLCO2B1	2,732760296	0,00225234
SPOCD1	2,629199572	4,54E-05	ARHGDIB	2,226199038	0,002446559
GAL	2,598934135	6,15E-05	PTHLH	4,711087434	0,002450317
PMEPA1	1,967218429	6,15E-05	IL6ST	2,123553616	0,002450317
SEMA7A	1,911459216	6,15E-05	F2RL1	1,701706227	0,002546264
SIM2	3,167334171	6,29E-05	CPA4	1,642588269	0,002547328
SERPINE1	2,798165062	6,57E-05	TRIML2	2,65612546	0,002803042
INHBA	4,186346592	7,54E-05	RRAD	2,356494017	0,002830075
GDF15	1,973998395	8,06E-05	CAV2	1,944528389	0,002907175
JUNB	1,952571749	8,06E-05	DUSP5	1,614895079	0,002978339
OXTR	2,296104211	8,32E-05	LTBP2	2,603423885	0,003245881
LAMC2	2,448525099	0,000121314	RAMP1	1,731342839	0,003662479
CRLF1	3,222009526	0,00013696	TAC1	1,590107658	0,003700528
AMIGO2	2,19537543	0,000188579	GPR87	2,290047196	0,003705592
POU2F2	1,920477198	0,000199134	EBI3	1,834331335	0,003890978
ANXA4	1,524080736	0,000203676	CLCA2	1,568271593	0,004203243
NGF	1,575477534	0,000214729	MX1	2,151667583	0,004673777
ADAM12	2,627168827	0,000241171	NPFFR2	2,071848324	0,004673777
PTPRN	1,898344697	0,000278524	FOSL1	2,033533552	0,004848003
TNFRSF11B	2,052407425	0,000306379	C1QL1	-1,518618864	0,00567038
FN1	2,097273737	0,000316043	CHRNA9	2,341930935	0,006317128
LDLRAD4	1,885583009	0,000366423	PROKR2	2,187361985	0,006317128
SPON2	1,664023037	0,000367141	CDKN2B	2,078064312	0,006317128
TES	1,638095057	0,000367141	C1QL3	2,251176908	0,006486544
LEFTY2	1,665034244	0,000405062	CLDN4	1,75335273	0,006486544
RAG1	3,975923848	0,000483132	MSTN	-1,615087924	0,006486544
MMP9	1,928522542	0,000499646	HSPA12A	1,709492127	0,006879139
PIF1	-1,50786107	0,000529838	CGB5	2,351065776	0,007003126
CXCL1	2,816696053	0,000539842	ANKRD1	1,667202737	0,00716927
NT5E	2,187868134	0,000665576	SRPX2	1,946225219	0,007347603
TAP1	1,706154724	0,000665576	GPR26	1,770766319	0,007671595
BHLHE40	2,552101744	0,000728008	COMP	1,563971118	0,007671595
IER3	1,535483982	0,00095399	GPR183	2,697987123	0,008448068
GLIPR1	2,359671334	0,001126099	CYGB	1,810473194	0,008551238

Table S3: Top DEGs, siSETDB1 *versus* siCTL during TGFβ response in WT myotubes

Genes	LogFC	Adj p-value	Genes	LogFC	Adj p-value	Genes	LogFC	Adj p-value
MAMDC2	-1,520498038	5,65E-05	ARSJ	-1,061549661	0,008451464	RPS6KL1	-1,267489634	0,023290191
SETDB1	-2,312260334	6,09E-05	FLT1	-1,332461615	0,008613958	CLCA2	-1,650785368	0,024689191
ACSL1	-1,455424424	0,000224437	HAS2	-1,247511382	0,008751755	KCNG3	-2,004431023	0,024951235
THBS1	-1,301394066	0,000224437	HMCN1	-1,044961141	0,008751755	APLNR	-2,306740676	0,027475948
BMPR2	-1,176640246	0,000224437	GPC6	-1,294102823	0,008794726	LAMA1	-1,105272513	0,028027906
ATP8B2	-1,05885524	0,000224437	ITGB6	-1,119913491	0,008794726	GSDMA	-1,111110953	0,028371421
PTGFRN	-1,53323687	0,000474681	LRP8	-1,042762389	0,008794726	GRK4	1,686838586	0,028644611
LIMA1	-1,175745808	0,000501383	ST8SIA2	-1,054476505	0,009159413	FREM1	-1,676226071	0,029134271
IGFBP3	-1,471141886	0,00057845	SCN11A	-1,758429933	0,009303076	MAN1A1	-1,378856426	0,029134271
TMED7	-1,443559104	0,00057845	DOK3	-1,010817943	0,009434878	NAALADL2	-1,525850019	0,029709578
TMEM109	-1,281138773	0,00057845	SUSD5	-1,206994107	0,009994918	LAMA2	-1,445964152	0,029886349
IGSF22	-1,179795818	0,00057845	MPZL2	-2,408914913	0,010148671	RPL13	1,177052671	0,030194496
NTM	-1,097552605	0,000596109	LRRN3	-2,423741639	0,010347444	LRRC2	-1,122755043	0,030247404
GRIK3	-1,382794973	0,000633272	DSCAML1	-1,583808697	0,010432176	PCDH18	-1,142379056	0,030682091
PRSS23	-1,21234796	0,000633272	HMOX1	1,01627009	0,010534753	CLDN22	-1,451975925	0,030829524
PARM1	-1,196752186	0,000723086	SLC39A11	-1,348927723	0,011182178	SCN2A	-1,301431103	0,031312572
RAMP2	-1,709990009	0,00072624	ENPP6	-1,329673444	0,011182178	DNAJC22	-1,866109729	0,031497413
PCDHA4	-1,179801452	0,000866001	SLC27A1	-1,039664553	0,011182178	PRKCQ	-1,748274946	0,031643005
SLC8A1	-2,168674612	0,001035925	KY	-1,136090745	0,011569641	TLL2	-1,382705171	0,031643005
IGSF3	-1,452586062	0,001260586	HOXA1	-1,869923761	0,011589371	HPDL	-1,0274529	0,031691539
UGGT2	-1,373806653	0,001260586	WNT8A	-1,723404066	0,011605214	CLGN	-1,00964704	0,031709166
CD109	-1,206613739	0,001260586	DCX	-1,172252082	0,011605214	SMARCAD1	-2,674991532	0,032096292
ZFYVE27	-1,146034744	0,001260586	CHRM3	-1,328414995	0,01184128	C1QL3	-1,110617004	0,032165845
AKR1C3	1,196726021	0,001288236	ARSB	-1,05339903	0,01184128	KIAA1549L	-1,301619635	0,032682881
ARHGAP26	-1,44235698	0,001370776	RAG1	-1,520711756	0,0120045	TMED8	-1,039140935	0,033560494
GPR137C	-1,350324674	0,001387303	LRFN2	-1,269349114	0,012103738	OAF	-1,149040279	0,033574106
FGF13	-1,003563077	0,001387303	NFIA	-1,56438338	0,01222235	ISM1	-1,131304626	0,033574106
NRP2	-1,303642909	0,001435207	SLC4A8	-1,423660225	0,012780684	LRRC37A	-1,179829418	0,033755698
OR2H2	-3,608774047	0,001483697	ITGA4	-1,093763889	0,012860251	PER3	-1,000955169	0,034677493
TMEM87B	-1,158116859	0,001779461	MMP25	-1,194320365	0,013314621	LRFN5	-1,607087431	0,035473366
SMTNL1	-1,069024474	0,001779461	OLFML1	-1,985558064	0,013852692	DPY19L2	-1,043176127	0,035841864
TAL2	-1,110677244	0,001884083	CNTNAP3	-1,246093716	0,014099073	MAST1	-1,282552641	0,036121551
CHDH	-1,49571412	0,002016351	CLDN1	-1,078499463	0,014200411	CCL4	1,885798209	0,037313963
PAK3	-1,059555572	0,002178155	CACNG4	-1,004577351	0,01422263	FAT3	-1,074630898	0,037313963
RIMS1	1,002106455	0,002207039	MKI67	-1,476461877	0,014255468	SPIB	-1,503325119	0,037761996
PLCD4	-1,709870369	0,002291852	MXRA8	-1,068346915	0,014255468	CDH4	-1,671716499	0,037870918
POLR3D	-1,05688535	0,002378671	GPLD1	-1,024911621	0,014268962	CBR1	-2,167825525	0,038060638
FRMPD1	-1,092657246	0,002688295	KCNK3	-1,637967552	0,014865901	ADAM33	-1,124290334	0,039108986
CACNA1E	-1,119064387	0,003649151	FAM71F1	1,003725779	0,015144667	UBE2Q2	-1,050446746	0,03956611
GRPR	-1,333005464	0,003905121	PAPPA	-1,235866455	0,015144667	DESI2	-1,177585114	0,040182971
SLC37A3	-1,035129517	0,003905121	GARNL3	-1,06505093	0,017145732	PODN	-1,010497684	0,040182971
PRIMA1	-2,433823631	0,004160811	RNF220	-1,073998499	0,017340312	GJC1	-1,132522857	0,041033645
DKK2	-1,476262445	0,004326207	ABCC9	-1,238756215	0,017696163	COL22A1	1,691834247	0,041291631
GAL3ST4	-1,296668125	0,004399025	CXCL1	-1,582775383	0,018516113	SCN4B	-1,181753524	0,042784027
ZNF107	-1,129045081	0,004399025	BRCA2	-1,44530328	0,018516113	HIST2H3D	-1,10116306	0,042784027
DPY19L3	-1,009527364	0,004399025	DOC2B	-1,133045331	0,018516113	YTHDC2	-1,074434231	0,044167886
KRT80	-1,272269317	0,005432878	THBD	-1,04413245	0,018516113	SLC6A3	1,32166898	0,044288292
TMEM182	-1,076767897	0,005750453	GAS2L1	-1,029502553	0,018516113	CNTN5	-1,150597818	0,044381061
CAMK4	-1,51980501	0,005834327	KDR	-1,412431822	0,019245021	SCN7A	-1,467305844	0,044542055
SPTLC3	-2,155845794	0,006288784	ROR1	-1,044621628	0,019603878	GRIN2A	-1,412058254	0,047051083
FSD1L	-1,190564652	0,006555192	PRDM8	-1,214084634	0,019779272	PCDH17	-1,143147116	0,049034992
KSR2	-1,802189364	0,00723519	VCAN	-1,192075825	0,019779272	TSPAN11	-1,52753589	0,049416366
SEZ6	-1,161203502	0,00723519	CDH6	-1,046088047	0,020401625	DNAJA4	-2,230979822	0,049955982
KIAA1211	-1,005076581	0,00723519	CNTN2	-1,109787194	0,020995772	FAXC	-1,046733564	0,049974705
APCDD1L	1,149403017	0,007527773	NEK10	-1,131980357	0,021155723			
ARNT2	-1,077264685	0,007527773	RRAD	1,152195301	0,021663764			
TENM3	-1,003246377	0,00807358	THEMIS2	-1,141961836	0,021663764			
TENM2	-1,01075277	0,008088306	SPTBN5	-1,200082097	0,022154881			
NT5E	-1,337136771	0,008215045	CARNS1	-1,189226427	0,022569992			

Table S4: Top DEGs, siSETDB1 *versus* siCTL during TGFβ response in DMD myotubes

Genes	LogFC	Adj p-value	Genes	LogFC	Adj p-value
MAMDC2	-1,637289048	1,59E-05	TBC1D30	-1,656334075	0,017074581
NTM	-1,190266731	0,000624532	TMEM109	-0,852260252	0,019233362
CACNG1	-1,183738436	0,001856085	MIXL1	-1,554596572	0,019233362
MAS1	-1,521127791	0,001856085	WNT8A	-2,267660056	0,020236735
IGFBP3	-1,376670225	0,00219827	RGS2	0,830392825	0,020236735
SLC8A1	-1,793845414	0,002530987	PRSS23	-0,872430004	0,021751043
ACSL1	-1,287676377	0,003389855	HEXB	-0,59923785	0,022743141
KLK4	1,787192625	0,003389855	OLIG3	-1,219844213	0,022795667
NT5E	-1,535868776	0,003691319	ANO2	-0,809761055	0,023330054
RAG1	-3,601343309	0,003691319	CXCL12	-1,052378458	0,023330054
KCNIP2	1,004157391	0,003691319	IL6ST	-1,816664708	0,023330054
ADAMTSL1	-1,233489406	0,003799356	DNAJA4	-1,462582406	0,0237548
ANTXR2	-0,898772223	0,004091009	C6orf118	-1,270419296	0,025251564
LRP8	-0,874823587	0,004452942	UCP2	0,591231944	0,025251564
FZD10	-2,477744095	0,010340651	FHIT	0,974006361	0,025251564
IFI16	-1,095725152	0,010354658	PCDHB5	-0,847006813	0,026603158
ADAMTS8	-0,749833348	0,010652676	MOXD1	-1,300307077	0,026603158
ITGA2	-0,833861933	0,010652676	NECAB3	0,57770924	0,026603158
ANKRD33B	1,309182508	0,010772045	MX1	-1,671442415	0,026673574
VEZT	-0,59658059	0,010948764	IGSF22	-0,898697771	0,027562876
LIMA1	-0,805583317	0,010948764	SETDB1	-1,0498379	0,028675644
ITGB6	-1,363673345	0,011201951	FBLN1	-0,829507714	0,02996258
POLN	-0,950024758	0,011211256	RPSA	-1,790736619	0,033731882
RAMP2	-1,299151192	0,011334712	MATN2	-0,849749358	0,035265666
NBPF3	-1,164405324	0,011446775	MLIP	-1,211291173	0,035922112
CANX	-0,679162696	0,012181954	PGPEP1	0,632953024	0,035922112
BMPR2	-0,753326146	0,012181954	SFMBT2	0,683128284	0,035922112
ZFYVE27	-0,809883445	0,012181954	RAP1GAP	0,71600913	0,035922112
NFE2L3	-0,939940084	0,012181954	NKAIN2	-1,137475403	0,039898636
KNDC1	0,708511646	0,012238511	MARCH2	-0,595011539	0,039948549
WNT5A	-0,703188943	0,013030024	KRT17	0,948663616	0,042060417
TMEM30A	-0,705417259	0,013030024	FRY	-0,58182675	0,042984885
THBS1	-0,73807264	0,013030024	PTGFRN	-0,743069331	0,042984885
NEK10	-1,269427146	0,013030024	RTL1	-0,890963897	0,042984885
PTPN21	-0,775583543	0,013160275	ARRDC2	-1,191091084	0,042984885
FAM20A	-0,869507338	0,013160275	AMHR2	-2,339438416	0,042984885
KIT	-0,917148702	0,013160275	GATM	0,521594077	0,042984885
PIEZO2	-1,18208656	0,013160275	FXYD4	3,180179625	0,042984885
SLITRK5	-1,053308863	0,015640425	ZFP28	-1,866547884	0,045979659
SYNDIG1	0,893507731	0,015866611	DDOST	-0,696025419	0,047060967
LIF	-0,657896325	0,017074581	PRDM8	-1,346055025	0,047060967
LRTM1	-0,907971475	0,017074581	SLA	1,287085305	0,048993455
